# Crystal structure of the 1:1 cocrystal 5,5′-(triaz-1-ene-1,3-di­yl)bis­(3-nitro-1*H*-1,2,4-triazole)–tri­ethylammonium nitrate

**DOI:** 10.1107/S205698902401096X

**Published:** 2024-11-22

**Authors:** Matthew Gettings, Matthias Zeller, Davin Piercey

**Affiliations:** a Department of Chemistry & Life Science United States Military Academy, Bartlett Hall West Point NY 10996 USA; b Department of Chemistry, Purdue University, 560 Oval Dr., West Lafayette, IN 47907, USA; c School of Materials Engineering, School of Mechanical Engineering, Purdue Energetics Research Center, Purdue University, 205 Gates Road, West Lafayette, IN 47907, USA; Illinois State University, USA

**Keywords:** crystal structure, 1,2,4-triazole, energetic material, hydrogen bonding, X-ray crystallography, high Z*, pseudosymmetry

## Abstract

The tri­ethyl­ammonium nitrate cocrystal of 5,5′-(triaz-1-ene-1,3-di­yl)bis­(3-nitro-1*H*-1,2,4-triazole), C_4_H_3_N_11_O_4_·C_6_H_16_N·NO_3_ (**3**), was obtained unintentionally as the product of an attempted synthesis of a heterocyclic *C*-bromo­nitrilimine from 3-amino-5-nitro-1,2,4-triazole (ANTA). In the solid state at 150 K, the structure of **3** is modulated by a pseudo-translation along [010] with Z* = 4 (*Z* = 8 in *P*1). Exact translational symmetry is broken by a slight modulation of the triazene mol­ecules and nitrate anions, and by disorder of two of the four tri­ethyl­ammonium cations. An extensive hydrogen-bonding network connects the components of the structure and enables a relatively high density of 1.516 g cm^−3^. This feature, as well as its high nitro­gen content, make this rare triazene-bridged 1,2,4-triazole and possible related similar compounds of inter­est as potential energetic materials.

## Abstract 2

The tri­ethyl­ammonium nitrate cocrystal of 5,5′-(triaz-1-ene-1,3-di­yl)bis­(3-nitro-1*H*-1,2,4-triazole), obtained unintentionally from 3-amino-5-nitro-1,2,4-triazole (ANTA), exhibits extensive hydrogen bonding and modulation by pseudo-translation with Z* = 4.

## 1. Chemical context

Several energetic materials and high-nitro­gen materials have been generated from heterocyclic *C*-bromo­nitrilimines based on the well-known 3-amino-5-nitro-1,2,4-triazole (ANTA) moiety (Gettings *et al.*, 2021 ▸; Thoenen *et al.*, 2022 ▸). The 1,2,4-triazole heterocycle contains two carbons, which enable addition of substituents such as C-amino and C-nitro to the backbone. Furthermore, these same carbons may form an exocyclic C—C bond, bridging two 1,2,4-triazoles together (Dippold & Klapötke, 2013 ▸). The bridged motifs may also be linked by nitro­gen chains including N—N (azo) (Yount *et al.*, 2020 ▸, 2021 ▸) and N=N—N (triazene) (Feng *et al.*, 2021 ▸; Jiang *et al.*, 2023 ▸).

Energetic materials such as 4,4′,5,5′-tetra­amino-3,3′-azo-bis-1,2,4-triazole (TAABT) and its nitrated derivative (DNDAABT) are azo-bridged 1,2,4-triazoles (Yount *et al.*, 2020 ▸, 2021 ▸). Azo-bridged triazoles are less toxic and have a lower environmental impact than most metal-based primary energetic materials such as lead azide (Türker, 2016 ▸). Other researchers studied azo- and triazene-bridged 1,2,3-triazoles, finding improved performance (thermal stability, insensitivity, and higher crystal density) of the azo-bridged analog compared to the triazene (Feng *et al.*, 2021 ▸).

**Figure 1:**
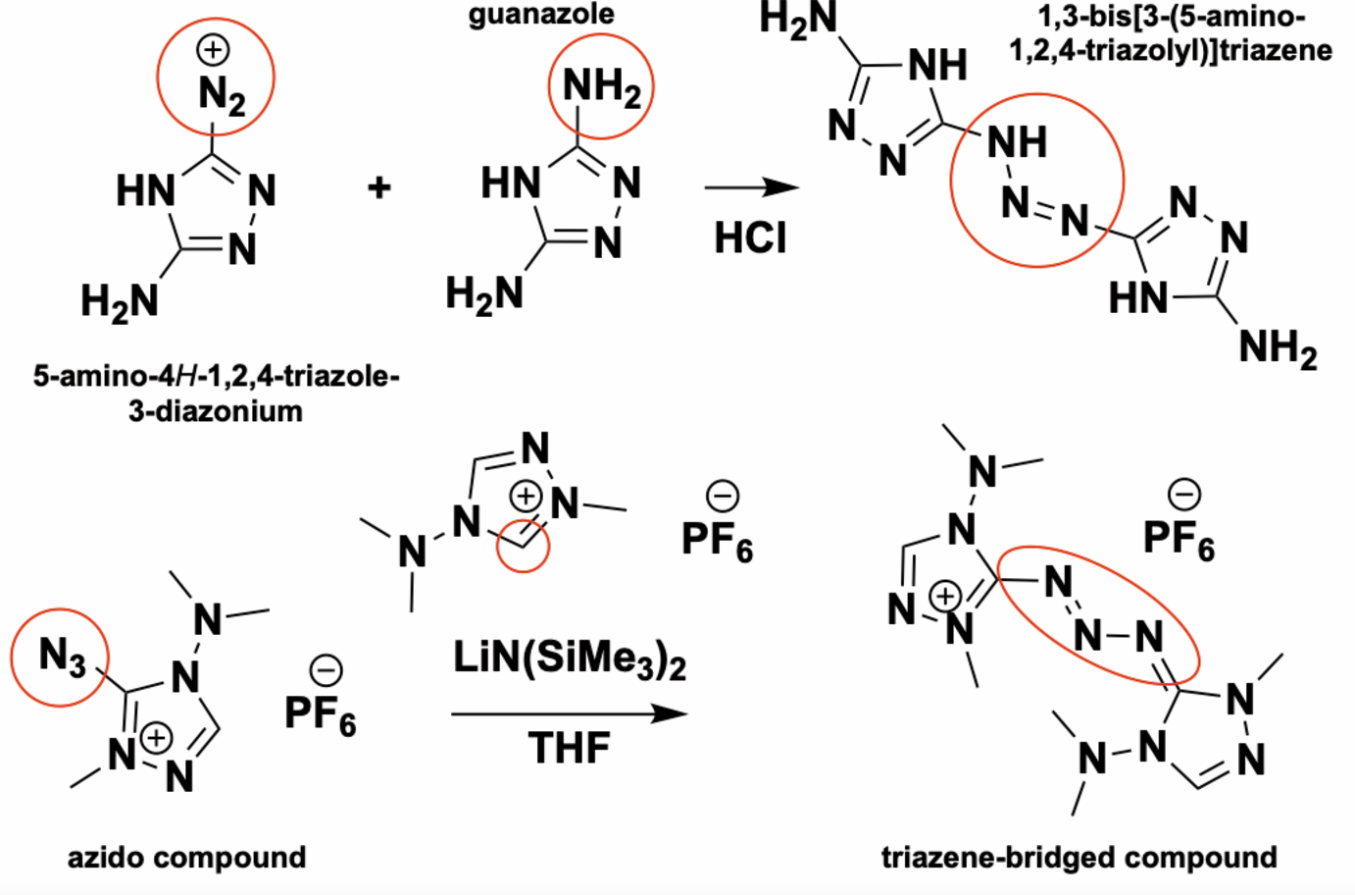
Triazene-bridged 1,2,4-triazoles obtained by reacting 5-amino-4*H*-1,2,4-triazole-3-diazo­nium with guanazole (top). Alternatively, the azido-substituted 1,2,4-triazole reacting with the carbene forms a triazene bridge (bottom).

There are a few known routes to effectively synthesize triazene-bridged triazoles. In an early synthesis, a diazo­nium solution prepared from 3-amino-5-nitro­samino-1,2,4-triazole treated with 3,5-di­amino-1,2,4-triazole (guanazole) formed 1,3-bis­[3-(5-amino-1,2,4-triazol­yl)]triazene (Hauser, 1964 ▸). In this reaction, the triazene bridge is formed by the diazo­nium of the first compound and amine of guanazole (Fig. 1 ▸, top). In another synthesis, 5-azido-4-(di­methyl­amino)-1-methyl-1,2,4-triazolium hexa­fluorido­phosphate reacts with the carbene of a triazolium salt to form the triazene bridge (Laus *et al.*, 2016 ▸) (Fig. 1 ▸, bottom). Similar nitro­gen-rich catenated structures featuring triazene-bridged 1,2,4-triazoles have been used as ligands coordinated with metal complexes (copper, palladium, and nickel; Hanot *et al.*, 1994 ▸, 1999 ▸). In a recent paper, Ma’s research group obtained both 5,5′-di­nitro-3,3′-triazene-1,2,4-triazole and 5-nitro-3,3′-triazene-1,2,4-triazole *via* diazo­nium-*N*-coupling reactions (Jiang *et al.*, 2023 ▸). From both of these triazene-bridged 1,2,4-triazoles, several more energetic salts (potassium, ammonium, hydrazinium, and hydroxyl­ammonium) were reported, demonstrating good sensitivities, thermal stabilities, and high calculated detonation properties (Jiang *et al.*, 2023 ▸).

In this manuscript we report a rare triazene-bridged nitro-1,2,4-triazole as a cocrystal with tri­ethyl­ammonium nitrate.
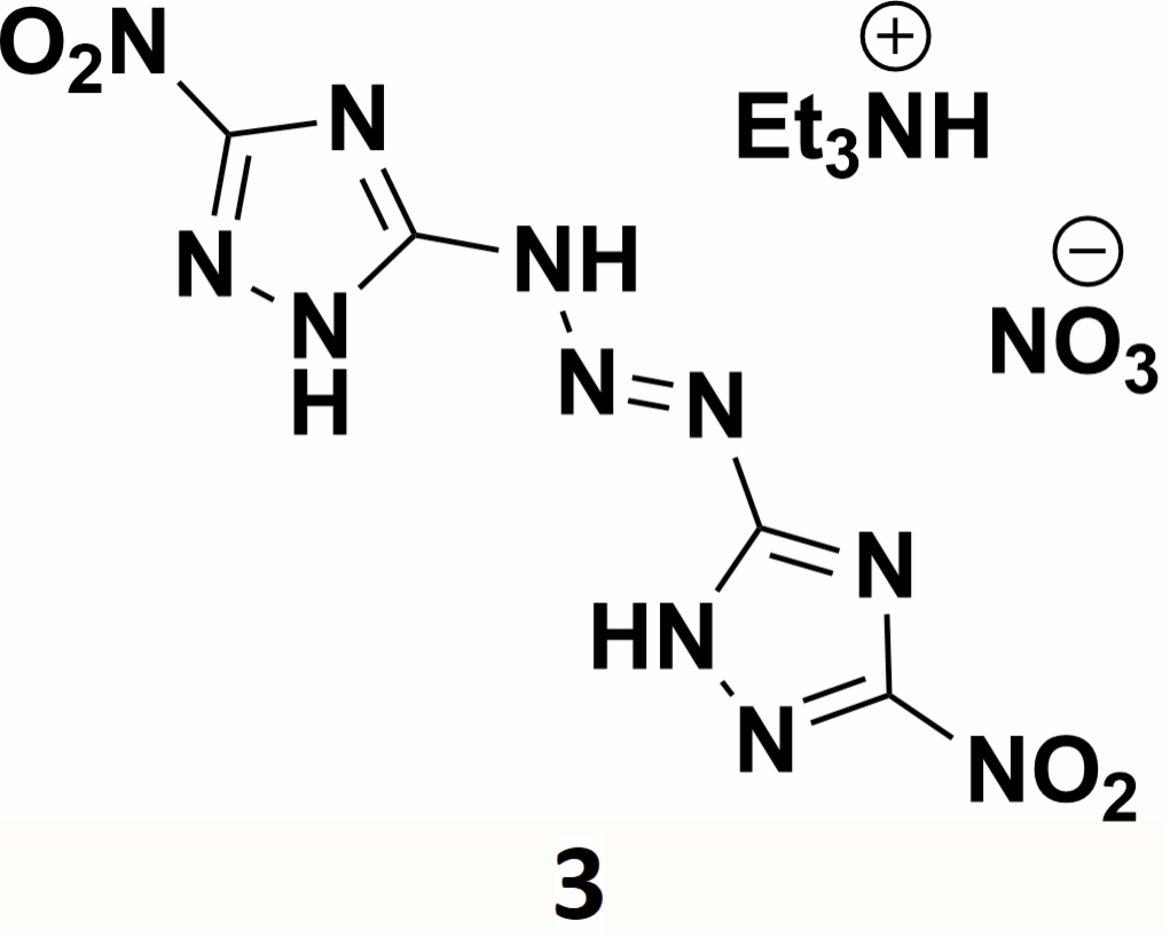


## 2. Structural commentary

**Figure 2:**
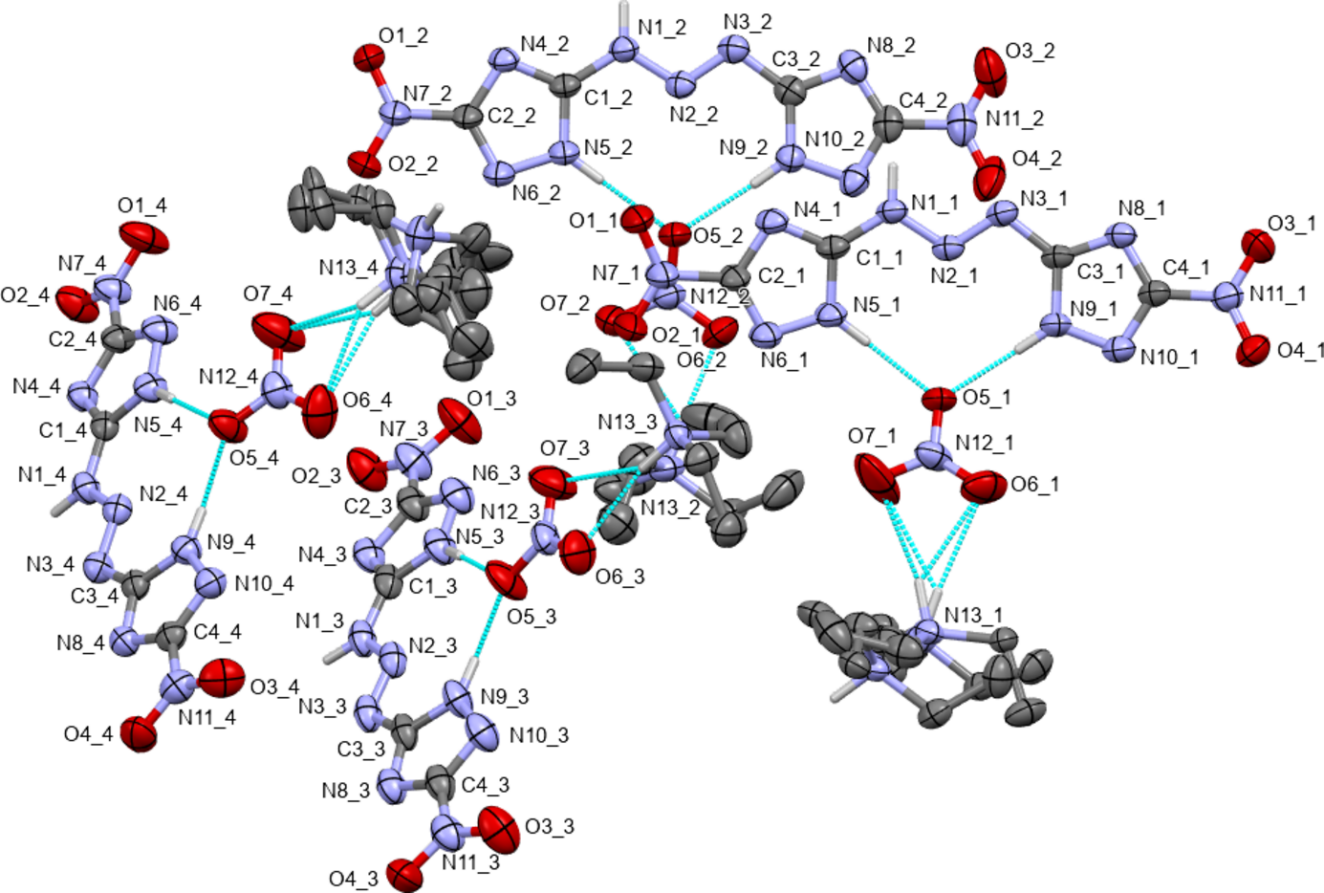
View of the asymmetric unit of the structure of the tri­ethyl­ammonium nitrate cocrystal of 5,5′-(triaz-1-ene-1,3-di­yl)bis­(3-nitro-1*H*-1,2,4-triazole) (**3**) with the labeling scheme. Ellipsoids are drawn at the 50% probability level. Carbon-bound H atoms as well as labels for the tri­ethyl­ammonium C and minor moiety N atoms have been omitted for clarity. Hydrogen bonds within the asymmetric unit are shown as turquoise dashed lines. Those to symmetry-generated atoms are omitted.

The title compound **3** is a cocrystal of the triazene and tri­ethyl­ammonium nitrate having a chemical composition of C_4_H_3_N_11_O_4_·C_6_H_16_N·NO_3_ and possessing one tri­ethyl­ammonium cation, one nitrate anion, and the triazene mol­ecule (Fig. 2 ▸). Compound **3** crystallizes in the triclinic system (space group *P*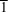) and four independent chemically identical copies of each of the constituent parts are present (*Z*′ = 4, *Z* = 8), with pseudo-translations along the *b*-axis direction. Exact translational symmetry is broken by a slight modulation of one of the triazene mol­ecule pairs and nitrate ions, and by disorder of some of the tri­ethyl­ammonium cations (see *Supra­molecular features* section for details). A common atom-numbering scheme was used for the four moieties, with residue numbers 1 through 4 used to distinguish between chemically equivalent atoms.

Each of the four triazene mol­ecules consists of two 5-nitro-1,2,4-triazole rings linked together by three catenated nitro­gen atoms (triazene) in a *trans* geometry. Each of the mol­ecules carries three acidic nitro­gen-bound hydrogen atoms, one at one of the triazene N atoms (N1), and one at each of the triazole rings (N5 and N9), thus rendering the mol­ecules charge neutral (all triazene H atoms were well resolved in difference-density maps and positions are also supported by hydrogen-bonding considerations). All four triazene mol­ecules are close to planar, with the largest deviations from planarity being observed for the nitro oxygen atoms. Root mean square deviations from planarity for all C and N atoms are 0.0718, 0.0589, 0.0877 and 0.0550 Å for mol­ecules 1 through 4, respectively. The largest deviation from planarity is observed for nitro oxygen O1 of residue 3 [0.406 (4) Å]. Bond distances and angles of the triazene mol­ecules are in the expected ranges, and agree with those of related triazenes such as the dihydrate of the triazene of title compound **3** (Jiang *et al.*, 2023 ▸). The N1—N2 bond length involving the protonated nitro­gen N1 with an average value of 1.336 Å is significantly (0.063 Å) longer than that of N2—N3 (1.273 Å), indicating localized single and double bonds for the triazene N_3_ units. Differences between the values for the four mol­ecules are insignificant [values in the four mol­ecules are N1—N2 = 1.333 (3), 1.334 (3), 1.339 (3) and 1.337 (3) Å; those for N2–N3 are 1.272 (3), 1.269 (3), 1.273 (3) and 1.275 (3) Å]. A similar trend is observed for the C—N bonds of the triazoles, but differences are smaller, as expected due to partial delocalization of the single and double bonds in a triazole. The C—N bond lengths involving the protonated N atoms N5 and N9 range from 1.340 (3) to 1.353 (3) Å (average 1.347 Å), those of unprotonated atoms N6 and N10 at 1.301 (3) to 1.317 (3) Å (average 1.312 Å) are slightly (0.035 Å) shorter. Other bond distances in the triazoles follow a similar trend and are as expected, and confirm the localized nature of the acidic hydrogen atoms. In the related tripotassium salt of the title compound triazole (Jiang *et al.*, 2023 ▸), which is fully deprotonated, bond distances differ much less. The triazene N—N bonds are virtually identical (1.301 and 1.304 Å), and all C—N bonds of the triazolate are clustered within a tight margin (1.324 to 1.353 Å).

**Figure 3:**
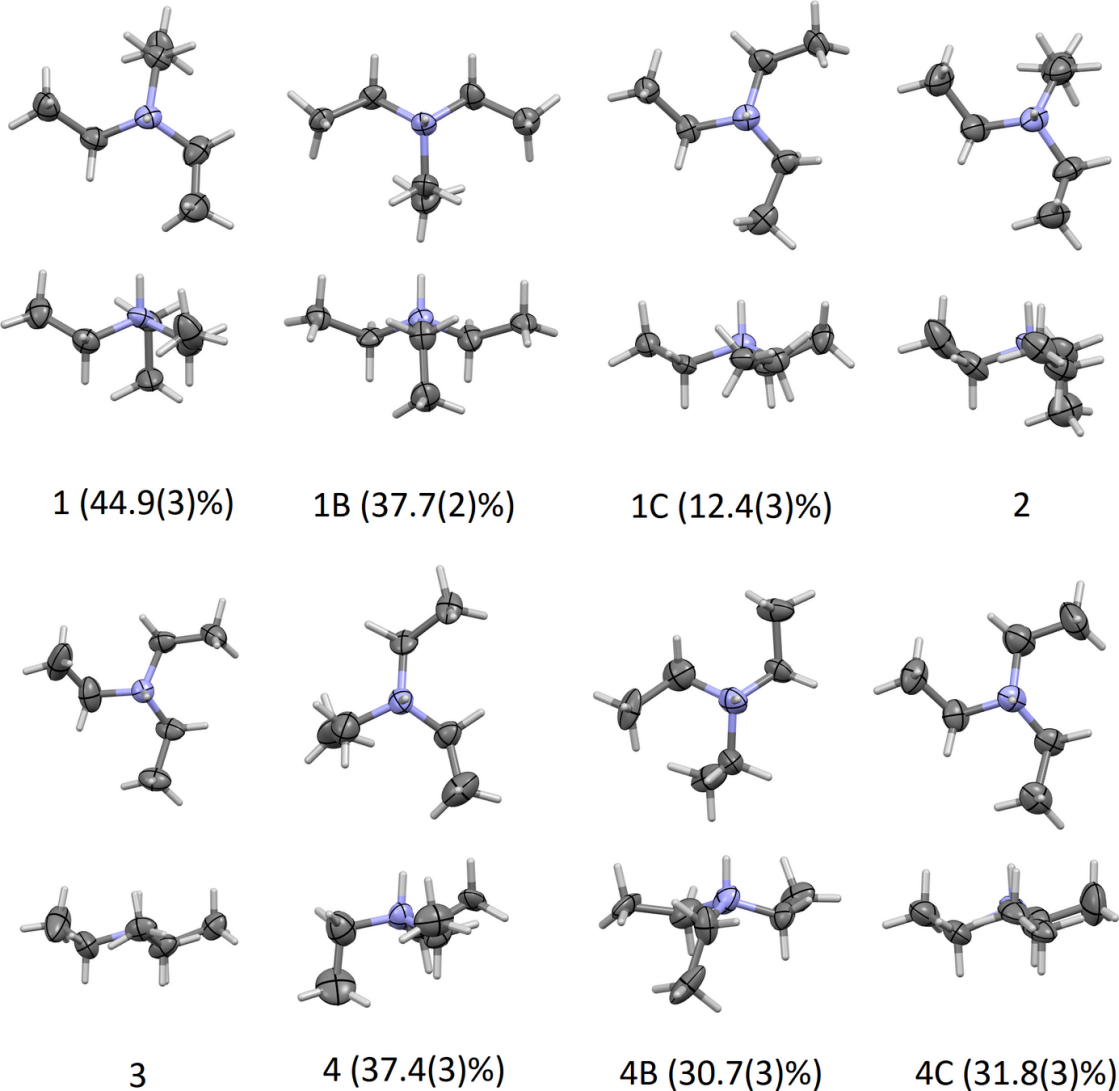
The various conformations of the tri­ethyl­ammonium cations. View along the N—H bond direction (top rows) and side-on views (bottom rows). The occupancy rates are given for disordered cations.

Bond distances and angles within the nitrate anions are unexceptional. Two of the four triethyl ammonium cations (cations 1 and 4) are each disordered over three orientations (see *Refinement* section for details of the refinement strategy). The disorder involves inversion at the ammonium N atom, and variation of the ethyl torsion angles. Three close-to-*trans* C—N—C—C torsion angles are observed for cation 3 (not disordered) as well as the third moiety of cations 1 and 4. One ethyl group is rotated into a *gauche* orientation (while the other two maintain *trans*), which is observed for the not-disordered cation 2 and the major moieties of 1 and 4, the second and third moieties of 4, and the third moiety of 3. Again the second moiety of 1 is different, featuring one *trans*, one *gauche* and one *anti* orientation (with the two non-*gauche* ethyl groups rotated in opposite directions). *Gauche-*oriented methyl groups also differ by pointing either up or down relative to the direction of the N—H bond. The different conformations of the cations are shown in Fig. 3 ▸, and representative torsion angles are given in Table 1 ▸.

## 3. Supra­molecular features

**Figure 4:**
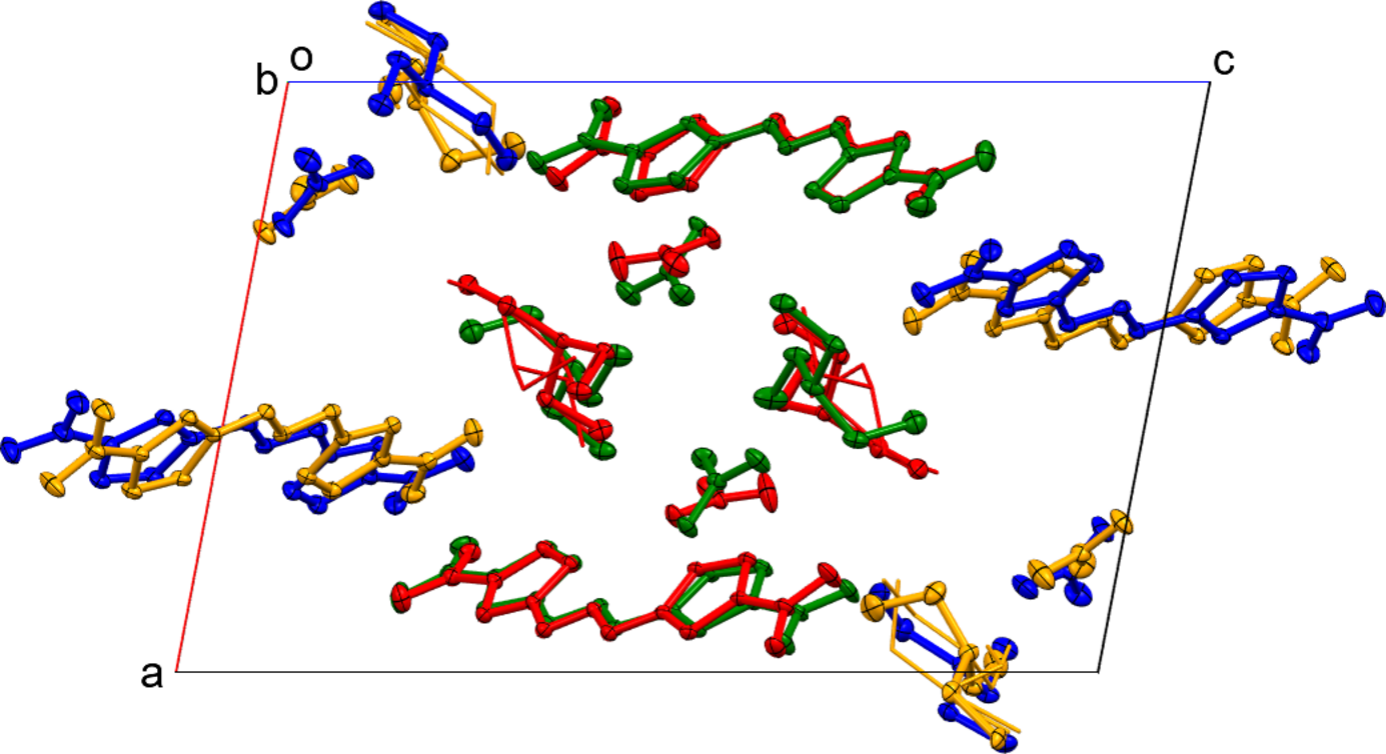
Modulation along the *b-*axis direction. Mol­ecules are color coded by residue numbers, with triazene mol­ecules and ions of residue 1 in red, of 2 in green, of 3 in blue and of 4 in dark yellow. Ellipsoids are drawn at the 20% probability level to better show modulation of atoms (minor moiety cations 1 and 4 are shown in stick mode).

The presence of four crystallographically independent repeat units warrants an investigation for the presence of pseudo-symmetry. Indeed, upon closer inspection a pseudo-translation becomes apparent that relates the components of the structure along the *b*-axis direction. When viewed down this direction, the components of residue 1 relate to those of residue 2, and those of 3 to those of 4. Translational symmetry is nearly perfectly obeyed for the triazene mol­ecules 1 and 2, while for mol­ecules 3 and 4 a slight shift by about half a bond length is observed (Fig. 4 ▸). The nitrate ions are also slightly modulated along [010]. For the cations, exact translational symmetry is also broken by the presence of disorder for cations 1 and 4, which is not present for the pseudotranslationally related cations 2 and 3. Exact translational symmetry is also absent when disorder is ignored, and only the most prevalent moieties are compared to each other. The cations are slightly shifted laterally with respect to each other, and modulated by differing torsion angles (see Table 1 ▸). Using default cutoff values *PLATON* (Spek, 2020 ▸) reports an 82% fit for translational symmetry along [010]. The absence of exact translational symmetry is also supported by the intensity of reflections affected by pseudotranslation, which are clearly observed. The average intensity of the satellite reflections is 4.8 (*I*/σ = 3.5), while the intensity for all reflections averages to 13.6 (*I*/σ = 4.6).

**Figure 5:**
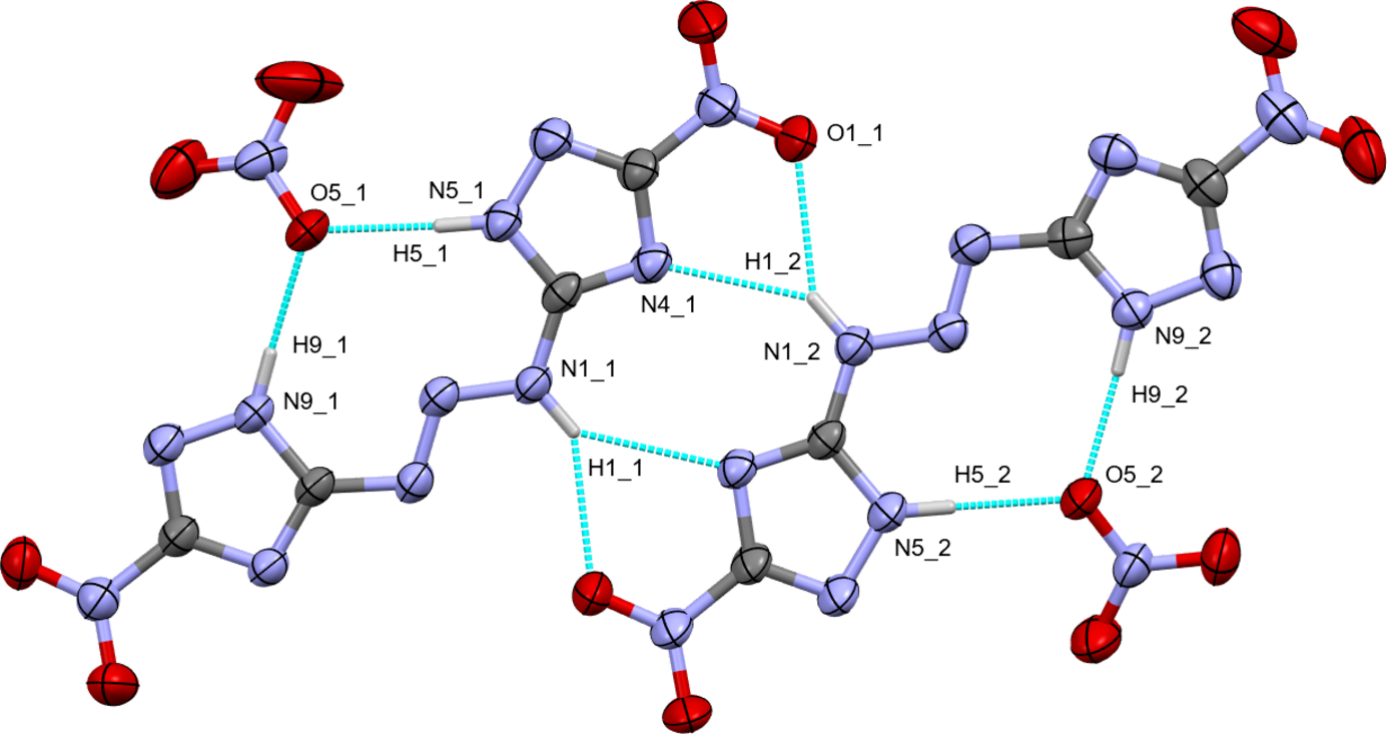
Dimers formed by bifurcated N—H⋯N hydrogen bonds between triazene mol­ecules, as well as the N—H⋯O-bonded nitrate anions. Only atoms involved in hydrogen bonding are labeled for clarity. Mol­ecules 3 and 4 form an equivalent dimer.

Directional intra­molecular inter­actions are dominated by N—H⋯O and N—H⋯N hydrogen bonds of various kinds (Table 2 ▸). The triazene N—H group forms a bifurcated set of hydrogen bonds to atoms N4 and O2 of a neighboring mol­ecule. A reciprocal set of hydrogen bonds is formed from the other triazene, thus creating a pseudo-inversion-symmetric dimer (Fig. 5 ▸). Mol­ecules connected by hydrogen bonds are, however, symmetry-independent and not related by actual inversion symmetry. The dimers are formed between mol­ecule 1 and mol­ecule 3 (at −*x*, 2 − *y*, 1 − *z*), and between mol­ecule 2 and mol­ecule 4 (also at −*x*, 2 − *y*, 1 − *z*).

Each of the triazenes is also hydrogen bonded *via* the tetra­zole N–H groups to atom O5 of one of the nitrate anions (Fig. 5 ▸). O5 acts as acceptor for two N–H⋯O hydrogen bonds from the same triazene-nitro­triazole mol­ecule. The nitrate ions thus bonded are nearly coplanar with the neutral mol­ecules, with only a slight tilt between their mean planes of 23.7 (2), 19.4 (2), 14.3 (2) and 17.1 (2)° for mol­ecule pairs 1 through 4, respectively.

Additional hydrogen bonds originate from the tri­ethyl­ammonium cations. These do, however, vary due to disorder of two of the four cations. The non-disordered cations of the major moieties as well as each minor moiety of the disordered cations do hydrogen bond in a bifurcated manner to O6 and O7 of the nitrate anions. Bonding parameters do vary with the hydrogen bonds to the second oxygen atom with some of the inter­actions being rather weak, rendering the hydrogen bonds nearly not bifurcated (see the hydrogen-bonding table for exact numerical values). The second moieties of both disordered cations are inverted at the nitro­gen atoms, thus breaking the hydrogen bond to the nitrate anions (weak C—H⋯O bonds are formed instead; see hydrogen-bonding Table 2 ▸). The ammonium N—H groups still form hydrogen bonds, but the acceptors are nitro­gen atoms of triazole rings: N8_4 at 1 − *x*, 1 − *y*, −*z* for cation 3B, and N10_3 at −*x*, 1 − *y*, −*z* for cation 1B.

The extensive hydrogen-bonding network facilitates a relatively high density of 1.516 g cm^−3^, but not quite as high as that of the dihydrate of 5,5′-(triaz-1-ene-1,3-di­yl)bis­(3-nitro-1*H*-1,2,4-triazole), which was reported as 1.765 g cm^−3^ (Jiang *et al.*, 2023 ▸). These high densities and the high-nitro­gen content make this triazene-bridged 1,2,4-triazole of inter­est as a potential future energetic material, which already prompted a recent investigation of the energetic properties of some of its derivatives (Jiang *et al.*, 2023 ▸).

## 4. Database survey

A structure search of the Cambridge Structural Database (CSD, v5.43, March 2022; Groom *et al.*, 2016 ▸) for an *R*—NH—N=N—*R* unit yielded 347 hits, about equally distributed between linear triazenes and cyclic 1,2,3-triazoles. The most closely related hits are that of the dihydrate and of the tripotassium salt 3.5 hydrate of the triazene of title compound **3** (CSD refcodes DIFYOK and DIFYUQ, CCDC 2225841 and 2225842; Jiang *et al.*, 2023 ▸). The dihydrate differs from the triazene in **3** by a rotation of one of the triazoles, which allows hydrogen bonding with a water mol­ecule, replacing the nitrate atom O5 in **3***via* one N—H⋯O and one O—H⋯N hydrogen bond. In the tripotassium salt both triazolates are rotated, and the solitary nitro­gen atom of the triazolates bond together with the middle triazene N atom to a potassium ion. The nitro groups both also inter­act weakly *via* one O atom with this potassium ion.

## 5. Synthesis and crystallization

**CAUTION!** The described compound **3** may be an energetic material with sensitivity to various stimuli. While we encountered no issues in the handling of this material, proper protective measures (face shield, ear protection, body armor, Kevlar gloves, and earthed equipment) should be used at all times.

A single crystal of the title compound was obtained unintentionally as the product of an attempted synthesis of a heterocyclic *C*-bromo­nitrilimine. 3-Amino-5-nitro-1,2,4-triazole (ANTA, **1**) was prepared according to the literature method (Manship *et al.*, 2020 ▸). An aqueous solution of ANTA (100 mg, 0.775 mmol) was cooled to 273–278 K. A separate chilled solution of sodium nitrite (62 mg) dissolved in water (5 mL) and nitric acid (0.06 mL, 15.8 *M*) was prepared. The acidic solution was added to the cold mixture with stirring, forming the highly unstable diazo­nium inter­mediate (**2**). The cold reaction mixture was stirred overnight and the next day, then tri­ethyl­amine (80 mg, 0.775 mmol) was added to the mixture with stirring for a few hours. The mixture was then set aside for slow evaporation. After several days, a mixture of larger block-shaped and smaller rod-shaped crystals was obtained. The block-shaped crystals were identified *via* single-crystal XRD as sodium nitrate [space group *P*
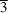
*c*1, *a* = 5.0650 (3), *c* = 16.5957 (17) Å]. The rod-shaped crystals were those of the title compound, a cocrystal of tri­ethyl­ammonium nitrate and 5,5′-(triaz-1-ene-1,3-di­yl)bis­(3-nitro-1*H*-1,2,4-triazole) (**3**). No other solid products could be identified and the material was not analyzed further.

## 6. Refinement

Crystal data, data collection and structure refinement details are summarized in Table 3 ▸.

Four crystallographically independent triazene mol­ecules and four nitrate-tri­ethyl­ammonium ion pairs are present in the crystal structure. A common atom-naming scheme was used for all four equivalent moieties, which are distinguished by their respective residue numbers (RESI 1 through 4). Two of the four tri­ethyl­ammonium cations are threefold disordered by being either hydrogen bonded to nitrate oxygen atoms, or to triazole nitro­gen atoms, and by different folding of their ethyl groups. All tri­ethyl­ammonium moieties were restrained to have similar geometries. *U*_ij_ components of the ADPs for disordered atoms closer to each other than 2.0 Å were restrained to be similar. Subject to these conditions, the occupancy rates refined to 0.499 (3), 0.377 (2) and 0.124 (3) for moieties A, B and C of residue 1, and 0.374 (3), 0.307 (3) and 0.318 (3) for moieties A, B and C of residue 4.

H atoms were positioned geometrically and constrained to ride on their parent atoms. C—H bond distances were constrained to 0.99 and 0.98 Å for aliphatic CH_2_ and CH_3_ moieties, respectively. N—H bond distances were constrained to 0.88 Å for planar (*sp*^2^-hybridized) and to 1.00 Å for ammonium *R*_3_N—H^+^ groups. Methyl CH_3_ groups were allowed to rotate but not to tip to best fit the experimental electron density. *U*_iso_(H) values were set to a multiple of *U*_eq_(C/N) (1.5 for CH_3_ and 1.2 for all other H atoms).

## Supplementary Material

Crystal structure: contains datablock(s) I. DOI: 10.1107/S205698902401096X/ej2009sup1.cif

Structure factors: contains datablock(s) I. DOI: 10.1107/S205698902401096X/ej2009Isup2.hkl

Supporting information file. DOI: 10.1107/S205698902401096X/ej2009Isup3.cml

CCDC reference: 2081094

Additional supporting information:  crystallographic information; 3D view; checkCIF report

## Figures and Tables

**Table 1:** Representative torsion angles (°) of the tri­ethyl­ammonium cations

	C5—N13—C7—C8	C7—N13—C9—C10	C9—N13—C5—C6
Cation 1-A	176.2 (7)	−62.6 (7)	169.7 (6)
Cation 1-B	58.9 (10)	−177.7 (7)	−59.1 (8)
Cation 1-C	172 (3)	171 (2)	−161 (2)
Cation 2	162.2 (2)	175.3 (3)	−58.5 (3)
Cation 3	173.0 (3)	174.6 (3)	172.5 (3)
Cation 4-A	170.0 (16)	−66.4 (13)	157.8 (14)
Cation 4-B	171.2 (17)	170.6 (14)	47.8 (13)
Cation 4-C	179.1 (13)	−176.2 (14)	−179.4 (16)

**Table 2:** Hydrogen-bond geometry (Å, °)

*D*—H⋯*A*	*D*—H	H⋯*A*	*D*⋯*A*	*D*—H⋯*A*
N1_1—H1_1⋯N4_2^i^	0.88	2.37	3.117 (3)	142
N1_1—H1_1⋯O1_2^i^	0.88	2.30	3.066 (3)	145
N5_1—H5_1⋯O5_1	0.88	1.87	2.745 (3)	173
N9_1—H9_1⋯O5_1	0.88	1.88	2.761 (3)	174
N13_1—H13_1⋯N12_1	1.00	2.60	3.565 (6)	162
N13_1—H13_1⋯O6_1	1.00	2.31	3.096 (6)	135
N13_1—H13_1⋯O7_1	1.00	2.26	3.249 (6)	169
C6_1—H6*A*_1⋯N8_4^ii^	0.98	2.69	3.650 (8)	168
C7_1—H7*B*_1⋯O4_4^ii^	0.99	2.55	3.387 (7)	142
C8_1—H8*B*_1⋯O6_2^iii^	0.98	2.58	3.518 (11)	161
C9_1—H9*B*_1⋯O6_2^iii^	0.99	2.44	3.222 (7)	136
N13*B*_1—H13*B*_1⋯N8_4^ii^	1.00	2.17	3.165 (6)	172
C5*B*_1—H5*C*_1⋯O2_3^iv^	0.99	2.64	3.629 (8)	173
C5*B*_1—H5*D*_1⋯N10_2^iii^	0.99	2.58	3.567 (7)	177
C7*B*_1—H7*D*_1⋯O6_1	0.99	2.62	3.547 (7)	156
C8*B*_1—H8*D*_1⋯O4_2^iii^	0.98	2.52	3.452 (11)	160
C9*B*_1—H9*D*_1⋯O2_2^iv^	0.99	2.53	3.146 (7)	120
C10*B*_1—H10*D*_1⋯O2_3^iv^	0.98	2.61	3.150 (8)	115
N13*C*_1—H13*C*_1⋯O6_1	1.00	2.29	3.27 (3)	167
N13*C*_1—H13*C*_1⋯O7_1	1.00	2.56	3.31 (2)	132
C5*C*_1—H5*F*_1⋯O2_2^iv^	0.99	2.55	3.32 (3)	135
C6*C*_1—H6*H*_1⋯N8_4^ii^	0.98	2.61	3.53 (3)	157
C6*C*_1—H6*I*_1⋯O7_1	0.98	2.52	3.34 (3)	141
C7*C*_1—H7*F*_1⋯O4_4^ii^	0.99	2.36	2.93 (2)	116
C9*C*_1—H9*F*_1⋯O6_2^iii^	0.99	2.33	3.20 (2)	147
N1_2—H1_2⋯N4_1^i^	0.88	2.35	3.096 (3)	143
N1_2—H1_2⋯O1_1^i^	0.88	2.27	3.035 (3)	145
N5_2—H5_2⋯O5_2	0.88	1.88	2.751 (3)	172
N9_2—H9_2⋯O5_2	0.88	1.89	2.770 (3)	177
N13_2—H13_2⋯N12_2	1.00	2.53	3.444 (3)	151
N13_2—H13_2⋯O6_2	1.00	1.96	2.933 (3)	164
N13_2—H13_2⋯O7_2	1.00	2.48	3.188 (3)	128
C5_2—H5*A*_2⋯O2_1	0.99	2.57	3.097 (4)	113
C9_2—H9*A*_2⋯O6_1^iii^	0.99	2.41	3.340 (5)	157
N1_3—H1_3⋯N4_4^v^	0.88	2.39	3.136 (3)	143
N1_3—H1_3⋯O2_4^v^	0.88	2.30	3.075 (3)	147
N5_3—H5_3⋯N12_3	0.88	2.70	3.523 (3)	157
N5_3—H5_3⋯O5_3	0.88	1.87	2.748 (4)	175
N9_3—H9_3⋯O5_3	0.88	1.86	2.743 (3)	178
N13_3—H13_3⋯N12_3	1.00	2.51	3.503 (3)	174
N13_3—H13_3⋯O6_3	1.00	2.14	3.068 (3)	153
N13_3—H13_3⋯O7_3	1.00	2.23	3.116 (3)	146
C5_3—H5*A*_3⋯O1_2^iv^	0.99	2.64	3.367 (4)	131
C7_3—H7*B*_3⋯O3_1^vi^	0.99	2.63	3.317 (4)	127
C9_3—H9*A*_3⋯O6_4^vii^	0.99	2.44	3.389 (4)	160
C9_3—H9*B*_3⋯O3_2^vi^	0.99	2.65	3.538 (4)	149
C10_3—H10*B*_3⋯O6_3	0.98	2.56	3.342 (4)	137
N1_4—H1_4⋯N4_3^v^	0.88	2.39	3.126 (3)	142
N1_4—H1_4⋯O2_3^v^	0.88	2.29	3.035 (3)	143
N5_4—H5_4⋯O5_4	0.88	1.86	2.732 (3)	169
N9_4—H9_4⋯O5_4	0.88	1.87	2.748 (3)	176
N13_4—H13_4⋯N12_4	1.00	2.65	3.505 (15)	144
N13_4—H13_4⋯O6_4	1.00	2.30	3.171 (16)	145
N13_4—H13_4⋯O7_4	1.00	2.34	3.019 (14)	124
C6_4—H6*A*_4⋯N10_3^vii^	0.98	2.59	3.52 (2)	157
C7_4—H7*A*_4⋯O3_1^vi^	0.99	2.63	3.410 (12)	136
C7_4—H7*B*_4⋯N10_3^vii^	0.99	2.68	3.394 (11)	129
C9_4—H9*A*_4⋯O1_3	0.99	2.62	3.203 (10)	118
C9_4—H9*B*_4⋯O7_4	0.99	2.53	3.096 (11)	116
C10_4—H10*A*_4⋯O1_1	0.98	2.59	3.158 (13)	117
C10_4—H10*B*_4⋯N6_2	0.98	2.54	3.219 (13)	126
N13*B*_4—H13*B*_4⋯N10_3^vii^	1.00	2.45	3.385 (9)	155
C5*B*_4—H5*C*_4⋯N8_2^i^	0.99	2.62	3.558 (11)	159
C5*B*_4—H5*D*_4⋯O1_1	0.99	2.54	3.286 (10)	132
C7*B*_4—H7*C*_4⋯O7_4	0.99	2.56	3.33 (2)	134
C7*B*_4—H7*D*_4⋯O3_2^i^	0.99	2.27	3.155 (17)	148
C9*B*_4—H9*C*_4⋯N12_4	0.99	2.70	3.666 (13)	166
C9*B*_4—H9*C*_4⋯O6_4	0.99	2.08	2.989 (12)	152
C9*B*_4—H9*C*_4⋯O7_4	0.99	2.64	3.540 (13)	151
C9*B*_4—H9*D*_4⋯O6_3^vii^	0.99	2.29	3.240 (13)	160
N13*C*_4—H13*C*_4⋯N12_4	1.00	2.63	3.626 (18)	176
N13*C*_4—H13*C*_4⋯O6_4	1.00	2.27	3.208 (18)	156
N13*C*_4—H13*C*_4⋯O7_4	1.00	2.31	3.208 (16)	149
C7*C*_4—H7*F*_4⋯O6_3^vii^	0.99	2.21	3.100 (13)	150
C8*C*_4—H8*I*_4⋯O6_4	0.98	2.63	3.48 (2)	146
C10*C*_4—H10*G*_4⋯O2_2	0.98	2.61	3.346 (17)	132
C10*C*_4—H10*H*_4⋯O1_3	0.98	2.56	3.18 (2)	122

Symmetry codes: (i) 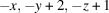; (ii) 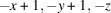; (iii) 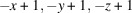; (iv) 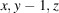; (v) 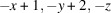; (vi) 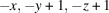; (vii) 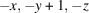.

**Table 3:** Experimental details

Crystal data	
Chemical formula	C_6_H_16_N^+^·NO_3_^−^·C_4_H_3_N_11_O_4_
*M*_r_	433.38
Crystal system, space group	Triclinic, *P* 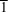
Temperature (K)	150
*a*, *b*, *c* (Å)	13.2412 (6), 14.3856 (6), 21.3601 (11)
α, β, γ (°)	108.186 (3), 99.785 (3), 91.272 (3)
*V* (Å^3^)	3796.9 (3)
*Z*	8
Radiation type	Mo *K*α
μ (mm^−1^)	0.13
Crystal size (mm)	0.23 × 0.22 × 0.20

Data collection	
Diffractometer	Bruker AXS D8 Quest diffractometer with PhotonII charge-integrating pixel array detector (CPAD)
Absorption correction	Multi-scan (*SADABS*; Krause *et al.*, 2015 ▸)
*T*_min_, *T*_max_	0.660, 0.746
No. of measured, independent and observed [*I* > 2σ(*I*)] reflections	58894, 18504, 10729
*R*_int_	0.078
(sin θ/λ)_max_ (Å^−1^)	0.667

Refinement	
*R*[*F*^2^ > 2σ(*F*^2^)], *wR*(*F*^2^), *S*	0.070, 0.222, 1.06
No. of reflections	18504
No. of parameters	1363
No. of restraints	1250
H-atom treatment	H-atom parameters constrained
Δρ_max_, Δρ_min_ (e Å^−3^)	0.54, −0.31

Computer programs: *APEX3* and *SAINT* (Bruker, 2020 ▸), *SHELXT* (Sheldrick, 2015*a* ▸), *SHELXL2019/2* (Sheldrick, 2015*b* ▸) and *ShelXle* (Hübschle *et al.*, 2011 ▸).

## supplementary crystallographic information

### 5,5'-(Triaz-1-ene-1,3-diyl)bis(3-nitro-1H-1,2,4-triazole)–triethylammonium nitrate (1/1) Crystal data

**Table:**

C_6_H_16_N^+^·NO_3_^−^·C_4_H_3_N_11_O_4_	*Z* = 8
*M**_r_* = 433.38	*F*(000) = 1808
Triclinic, *P*1	*D*_x_ = 1.516 Mg m^−^^3^
*a* = 13.2412 (6) Å	Mo *K*α radiation, λ = 0.71073 Å
*b* = 14.3856 (6) Å	Cell parameters from 9990 reflections
*c* = 21.3601 (11) Å	θ = 2.5–28.1°
α = 108.186 (3)°	µ = 0.13 mm^−^^1^
β = 99.785 (3)°	*T* = 150 K
γ = 91.272 (3)°	Rod, colourless
*V* = 3796.9 (3) Å^3^	0.23 × 0.22 × 0.20 mm

### 5,5'-(Triaz-1-ene-1,3-diyl)bis(3-nitro-1H-1,2,4-triazole)–triethylammonium nitrate (1/1) Data collection

**Table:**

Bruker AXS D8 Quest diffractometer with PhotonII charge-integrating pixel array detector (CPAD)	18504 independent reflections
Radiation source: fine focus sealed tube X-ray source	10729 reflections with *I* > 2σ(*I*)
Triumph curved graphite crystal monochromator	*R*_int_ = 0.078
Detector resolution: 7.4074 pixels mm^-1^	θ_max_ = 28.3°, θ_min_ = 2.1°
ω and phi scans	*h* = −17→17
Absorption correction: multi-scan (SADABS; Krause *et al.*, 2015)	*k* = −18→19
*T*_min_ = 0.660, *T*_max_ = 0.746	*l* = −28→28
58894 measured reflections	

### 5,5'-(Triaz-1-ene-1,3-diyl)bis(3-nitro-1H-1,2,4-triazole)–triethylammonium nitrate (1/1) Refinement

**Table:**

Refinement on *F*^2^	Primary atom site location: dual
Least-squares matrix: full	Secondary atom site location: difference Fourier map
*R*[*F*^2^ > 2σ(*F*^2^)] = 0.070	Hydrogen site location: inferred from neighbouring sites
*wR*(*F*^2^) = 0.222	H-atom parameters constrained
*S* = 1.06	*w* = 1/[σ^2^(*F*_o_^2^) + (0.1054*P*)^2^ + 0.9597*P*] where *P* = (*F*_o_^2^ + 2*F*_c_^2^)/3
18504 reflections	(Δ/σ)_max_ < 0.001
1363 parameters	Δρ_max_ = 0.54 e Å^−^^3^
1250 restraints	Δρ_min_ = −0.31 e Å^−^^3^

### 5,5'-(Triaz-1-ene-1,3-diyl)bis(3-nitro-1H-1,2,4-triazole)–triethylammonium nitrate (1/1) Special details

**Table:**

Geometry. All esds (except the esd in the dihedral angle between two l.s. planes) are estimated using the full covariance matrix. The cell esds are taken into account individually in the estimation of esds in distances, angles and torsion angles; correlations between esds in cell parameters are only used when they are defined by crystal symmetry. An approximate (isotropic) treatment of cell esds is used for estimating esds involving l.s. planes.

### 5,5'-(Triaz-1-ene-1,3-diyl)bis(3-nitro-1H-1,2,4-triazole)–triethylammonium nitrate (1/1) Fractional atomic coordinates and isotropic or equivalent isotropic displacement parameters (Å^2^)

**Table:**

	*x*	*y*	*z*	*U*_iso_*/*U*_eq_	Occ. (<1)
N1_1	0.06591 (15)	0.63088 (15)	0.53859 (10)	0.0359 (5)	
H1_1	0.020391	0.667591	0.557855	0.043*	
N2_1	0.10535 (14)	0.55936 (15)	0.56000 (10)	0.0339 (4)	
N3_1	0.07098 (15)	0.54858 (15)	0.60962 (11)	0.0364 (5)	
N4_1	0.06680 (14)	0.70492 (16)	0.45249 (11)	0.0374 (5)	
N5_1	0.17397 (15)	0.59196 (16)	0.45651 (11)	0.0389 (5)	
H5_1	0.206643	0.547480	0.470543	0.047*	
N6_1	0.18970 (16)	0.61876 (18)	0.40342 (11)	0.0435 (5)	
N7_1	0.10983 (17)	0.7324 (2)	0.35323 (13)	0.0514 (6)	
N8_1	0.09671 (15)	0.44356 (16)	0.67780 (10)	0.0367 (5)	
N9_1	0.18260 (15)	0.41189 (15)	0.59504 (11)	0.0368 (5)	
H9_1	0.205819	0.417354	0.560007	0.044*	
N10_1	0.20728 (16)	0.34321 (16)	0.62469 (11)	0.0411 (5)	
N11_1	0.16077 (17)	0.31206 (17)	0.72019 (12)	0.0427 (5)	
C1_1	0.10078 (16)	0.64374 (18)	0.48451 (12)	0.0340 (5)	
C2_1	0.12441 (17)	0.6852 (2)	0.40431 (13)	0.0394 (6)	
C3_1	0.11689 (17)	0.47100 (18)	0.62719 (12)	0.0339 (5)	
C4_1	0.15424 (18)	0.36733 (18)	0.67334 (13)	0.0366 (5)	
O1_1	0.04217 (16)	0.78886 (18)	0.35517 (11)	0.0605 (6)	
O2_1	0.1643 (2)	0.7132 (2)	0.31131 (14)	0.0821 (9)	
O3_1	0.1253 (2)	0.34661 (17)	0.77089 (11)	0.0636 (6)	
O4_1	0.20139 (15)	0.23458 (15)	0.70595 (11)	0.0514 (5)	
N12_1	0.29305 (18)	0.37193 (18)	0.44777 (12)	0.0479 (6)	
O5_1	0.26425 (16)	0.44069 (16)	0.49126 (11)	0.0549 (5)	
O6_1	0.3096 (2)	0.29387 (17)	0.45813 (14)	0.0731 (7)	
O7_1	0.3044 (3)	0.3842 (2)	0.39531 (15)	0.1171 (13)	
N13_1	0.4386 (3)	0.1963 (4)	0.3506 (3)	0.0369 (10)	0.499 (3)
H13_1	0.393260	0.249286	0.368090	0.044*	0.499 (3)
C5_1	0.3794 (5)	0.1295 (5)	0.2836 (3)	0.0544 (15)	0.499 (3)
H5A_1	0.424068	0.079078	0.262905	0.065*	0.499 (3)
H5B_1	0.319174	0.095418	0.291238	0.065*	0.499 (3)
C6_1	0.3440 (6)	0.1841 (8)	0.2372 (4)	0.071 (2)	0.499 (3)
H6A_1	0.403001	0.205446	0.221425	0.107*	0.499 (3)
H6B_1	0.310716	0.241667	0.260400	0.107*	0.499 (3)
H6C_1	0.294760	0.141562	0.198741	0.107*	0.499 (3)
C7_1	0.5369 (5)	0.2465 (5)	0.3447 (3)	0.0436 (12)	0.499 (3)
H7A_1	0.583921	0.196147	0.327355	0.052*	0.499 (3)
H7B_1	0.520465	0.282528	0.312067	0.052*	0.499 (3)
C8_1	0.5899 (9)	0.3167 (9)	0.4107 (6)	0.054 (2)	0.499 (3)
H8A_1	0.645142	0.356538	0.403560	0.080*	0.499 (3)
H8B_1	0.619098	0.279823	0.440156	0.080*	0.499 (3)
H8C_1	0.540245	0.359664	0.431669	0.080*	0.499 (3)
C9_1	0.4560 (4)	0.1406 (4)	0.4000 (3)	0.0368 (11)	0.499 (3)
H9A_1	0.388894	0.113430	0.403946	0.044*	0.499 (3)
H9B_1	0.487935	0.186471	0.444661	0.044*	0.499 (3)
C10_1	0.5241 (5)	0.0581 (4)	0.3799 (4)	0.0534 (15)	0.499 (3)
H10A_1	0.493201	0.012706	0.335564	0.080*	0.499 (3)
H10B_1	0.531576	0.022870	0.412778	0.080*	0.499 (3)
H10C_1	0.591915	0.084902	0.378189	0.080*	0.499 (3)
N13B_1	0.4824 (4)	0.2113 (4)	0.3040 (3)	0.0353 (11)	0.377 (2)
H13B_1	0.519120	0.222193	0.269315	0.042*	0.377 (2)
C5B_1	0.5202 (5)	0.1203 (5)	0.3186 (4)	0.0432 (14)	0.377 (2)
H5C_1	0.509761	0.065045	0.276010	0.052*	0.377 (2)
H5D_1	0.595000	0.131844	0.336706	0.052*	0.377 (2)
C6B_1	0.4677 (8)	0.0915 (8)	0.3673 (5)	0.057 (2)	0.377 (2)
H6D_1	0.396195	0.067584	0.346324	0.085*	0.377 (2)
H6E_1	0.469064	0.148640	0.407274	0.085*	0.377 (2)
H6F_1	0.503378	0.039473	0.380224	0.085*	0.377 (2)
C7B_1	0.5047 (5)	0.3014 (5)	0.3643 (3)	0.0367 (14)	0.377 (2)
H7C_1	0.477667	0.357806	0.351538	0.044*	0.377 (2)
H7D_1	0.467486	0.292369	0.398731	0.044*	0.377 (2)
C8B_1	0.6158 (8)	0.3255 (12)	0.3945 (5)	0.040 (2)	0.377 (2)
H8D_1	0.655807	0.318029	0.358844	0.060*	0.377 (2)
H8E_1	0.638379	0.280922	0.419755	0.060*	0.377 (2)
H8F_1	0.626480	0.393358	0.424790	0.060*	0.377 (2)
C9B_1	0.3699 (5)	0.1979 (7)	0.2758 (5)	0.0440 (16)	0.377 (2)
H9C_1	0.349652	0.255826	0.262418	0.053*	0.377 (2)
H9D_1	0.331869	0.195207	0.311410	0.053*	0.377 (2)
C10B_1	0.3382 (5)	0.1064 (6)	0.2160 (3)	0.0418 (16)	0.377 (2)
H10D_1	0.347229	0.048309	0.230404	0.063*	0.377 (2)
H10E_1	0.380975	0.104693	0.182515	0.063*	0.377 (2)
H10F_1	0.265832	0.106953	0.196301	0.063*	0.377 (2)
N13C_1	0.4649 (15)	0.2126 (14)	0.3527 (12)	0.040 (2)	0.124 (3)
H13C_1	0.425246	0.246662	0.387991	0.048*	0.124 (3)
C5C_1	0.389 (2)	0.1675 (18)	0.2881 (14)	0.044 (3)	0.124 (3)
H5E_1	0.419836	0.112899	0.258522	0.053*	0.124 (3)
H5F_1	0.327228	0.139225	0.298501	0.053*	0.124 (3)
C6C_1	0.355 (2)	0.237 (2)	0.2504 (13)	0.049 (4)	0.124 (3)
H6G_1	0.286854	0.213545	0.222380	0.074*	0.124 (3)
H6H_1	0.404313	0.241646	0.221989	0.074*	0.124 (3)
H6I_1	0.351035	0.302513	0.282401	0.074*	0.124 (3)
C7C_1	0.5413 (19)	0.2879 (17)	0.3480 (12)	0.038 (3)	0.124 (3)
H7E_1	0.585019	0.254168	0.315929	0.046*	0.124 (3)
H7F_1	0.503187	0.334590	0.329331	0.046*	0.124 (3)
C8C_1	0.609 (3)	0.344 (3)	0.4121 (18)	0.043 (6)	0.124 (3)
H8G_1	0.661354	0.385479	0.403359	0.065*	0.124 (3)
H8H_1	0.642520	0.298933	0.433352	0.065*	0.124 (3)
H8I_1	0.567796	0.386187	0.442050	0.065*	0.124 (3)
C9C_1	0.5232 (14)	0.1369 (15)	0.3763 (12)	0.044 (3)	0.124 (3)
H9E_1	0.559129	0.098657	0.340721	0.052*	0.124 (3)
H9F_1	0.575862	0.170770	0.416452	0.052*	0.124 (3)
C10C_1	0.4540 (19)	0.0684 (17)	0.3931 (15)	0.051 (4)	0.124 (3)
H10G_1	0.438732	0.006901	0.355556	0.077*	0.124 (3)
H10H_1	0.389791	0.098604	0.401213	0.077*	0.124 (3)
H10I_1	0.487962	0.054856	0.433398	0.077*	0.124 (3)
N1_2	0.07478 (15)	1.12408 (15)	0.53235 (10)	0.0357 (4)	
H1_2	0.034909	1.165760	0.553889	0.043*	
N2_2	0.11162 (14)	1.05149 (15)	0.55370 (10)	0.0342 (4)	
N3_2	0.08130 (15)	1.04713 (16)	0.60606 (11)	0.0380 (5)	
N4_2	0.07065 (14)	1.19234 (15)	0.44277 (10)	0.0345 (4)	
N5_2	0.16627 (15)	1.06873 (17)	0.44163 (11)	0.0409 (5)	
H5_2	0.196614	1.022469	0.454839	0.049*	
N6_2	0.17604 (16)	1.08954 (18)	0.38498 (12)	0.0444 (5)	
N7_2	0.10106 (16)	1.20785 (18)	0.33730 (12)	0.0459 (6)	
N8_2	0.10624 (16)	0.94789 (17)	0.67810 (11)	0.0428 (5)	
N9_2	0.18449 (15)	0.90478 (16)	0.59165 (11)	0.0384 (5)	
H9_2	0.205640	0.905982	0.554996	0.046*	
N10_2	0.20848 (16)	0.83875 (17)	0.62341 (12)	0.0430 (5)	
N11_2	0.16686 (19)	0.8182 (2)	0.72367 (13)	0.0540 (6)	
C1_2	0.10329 (16)	1.12954 (18)	0.47425 (12)	0.0333 (5)	
C2_2	0.11775 (17)	1.1624 (2)	0.38938 (13)	0.0385 (6)	
C3_2	0.12353 (18)	0.96862 (19)	0.62409 (12)	0.0371 (5)	
C4_2	0.16020 (19)	0.8689 (2)	0.67399 (14)	0.0422 (6)	
O1_2	0.04829 (17)	1.27760 (16)	0.34627 (11)	0.0563 (6)	
O2_2	0.13748 (18)	1.17290 (18)	0.28670 (12)	0.0655 (7)	
O3_2	0.1258 (2)	0.8522 (2)	0.77171 (13)	0.0792 (8)	
O4_2	0.21214 (18)	0.7430 (2)	0.71321 (14)	0.0768 (8)	
N12_2	0.32241 (16)	0.87547 (16)	0.45266 (12)	0.0403 (5)	
O5_2	0.24569 (15)	0.91202 (15)	0.47540 (11)	0.0515 (5)	
O6_2	0.36119 (17)	0.80825 (16)	0.47090 (12)	0.0616 (6)	
O7_2	0.35823 (19)	0.90722 (18)	0.41271 (13)	0.0689 (7)	
N13_2	0.48208 (16)	0.71740 (16)	0.36795 (13)	0.0470 (6)	
H13_2	0.451695	0.758249	0.406294	0.056*	
C5_2	0.3940 (2)	0.6772 (2)	0.31061 (18)	0.0573 (8)	
H5A_2	0.340801	0.644046	0.325488	0.069*	
H5B_2	0.363119	0.732486	0.298079	0.069*	
C6_2	0.4232 (3)	0.6069 (3)	0.25064 (19)	0.0724 (10)	
H6A_2	0.363730	0.588534	0.213739	0.109*	
H6B_2	0.445824	0.548150	0.261040	0.109*	
H6C_2	0.479287	0.637576	0.237433	0.109*	
C7_2	0.5563 (2)	0.7860 (2)	0.35432 (16)	0.0519 (7)	
H7A_2	0.599159	0.747135	0.323461	0.062*	
H7B_2	0.517283	0.828296	0.331839	0.062*	
C8_2	0.6247 (2)	0.8493 (2)	0.41722 (19)	0.0628 (9)	
H8A_2	0.667128	0.807929	0.437934	0.094*	
H8B_2	0.582523	0.886327	0.448511	0.094*	
H8C_2	0.669264	0.895121	0.406377	0.094*	
C9_2	0.5357 (2)	0.6403 (2)	0.39176 (19)	0.0569 (8)	
H9A_2	0.590528	0.672838	0.430934	0.068*	
H9B_2	0.568759	0.598837	0.355714	0.068*	
C10_2	0.4637 (3)	0.5756 (3)	0.4113 (2)	0.0803 (12)	
H10A_2	0.424912	0.616692	0.443202	0.120*	
H10B_2	0.503751	0.532887	0.432057	0.120*	
H10C_2	0.415937	0.535268	0.371152	0.120*	
N1_3	0.42161 (16)	0.59655 (18)	−0.02713 (12)	0.0445 (5)	
H1_3	0.461132	0.620267	−0.048687	0.053*	
N2_3	0.38328 (15)	0.50272 (18)	−0.04974 (12)	0.0428 (5)	
N3_3	0.41058 (16)	0.45159 (19)	−0.10362 (12)	0.0449 (5)	
N4_3	0.43110 (16)	0.74544 (19)	0.06459 (12)	0.0463 (6)	
N5_3	0.33233 (17)	0.6203 (2)	0.06451 (13)	0.0509 (6)	
H5_3	0.299832	0.561416	0.050406	0.061*	
N6_3	0.32615 (18)	0.6907 (2)	0.12217 (14)	0.0543 (6)	
N7_3	0.4064 (2)	0.8541 (2)	0.17197 (14)	0.0605 (7)	
N8_3	0.38474 (17)	0.28398 (19)	−0.17450 (12)	0.0469 (6)	
N9_3	0.30718 (18)	0.31847 (19)	−0.08839 (12)	0.0498 (6)	
H9_3	0.285985	0.353667	−0.051991	0.060*	
N10_3	0.28371 (19)	0.2216 (2)	−0.11856 (13)	0.0543 (6)	
N11_3	0.32564 (18)	0.1083 (2)	−0.21709 (13)	0.0525 (6)	
C1_3	0.39576 (18)	0.6534 (2)	0.03144 (15)	0.0440 (6)	
C2_3	0.3862 (2)	0.7620 (2)	0.11874 (15)	0.0492 (7)	
C3_3	0.36793 (19)	0.3543 (2)	−0.12184 (14)	0.0436 (6)	
C4_3	0.3317 (2)	0.2063 (2)	−0.16954 (15)	0.0484 (7)	
O1_3	0.3756 (2)	0.8628 (2)	0.22487 (13)	0.0822 (8)	
O2_3	0.45673 (19)	0.91903 (18)	0.16203 (12)	0.0705 (7)	
O3_3	0.28605 (19)	0.04050 (19)	−0.20312 (12)	0.0718 (7)	
O4_3	0.3611 (2)	0.09774 (18)	−0.26727 (13)	0.0700 (7)	
N12_3	0.17137 (18)	0.4149 (2)	0.05267 (13)	0.0490 (6)	
O5_3	0.24180 (19)	0.43224 (18)	0.02420 (13)	0.0723 (7)	
O6_3	0.1325 (2)	0.33120 (19)	0.03756 (14)	0.0758 (7)	
O7_3	0.1440 (2)	0.4814 (2)	0.09656 (15)	0.0825 (8)	
N13_3	0.01094 (15)	0.35366 (16)	0.15087 (10)	0.0369 (5)	
H13_3	0.060601	0.367247	0.123769	0.044*	
C5_3	0.0710 (3)	0.3627 (3)	0.21891 (17)	0.0715 (10)	
H5A_3	0.022693	0.353154	0.247418	0.086*	
H5B_3	0.118144	0.309517	0.214306	0.086*	
C6_3	0.1319 (3)	0.4579 (4)	0.2532 (2)	0.1016 (17)	
H6A_3	0.181426	0.452864	0.291428	0.152*	
H6B_3	0.085802	0.508878	0.269046	0.152*	
H6C_3	0.168803	0.475036	0.221718	0.152*	
C7_3	−0.0681 (2)	0.4273 (2)	0.15365 (15)	0.0500 (7)	
H7A_3	−0.120441	0.412733	0.178026	0.060*	
H7B_3	−0.034265	0.493430	0.179645	0.060*	
C8_3	−0.1210 (2)	0.4292 (2)	0.08657 (17)	0.0551 (8)	
H8A_3	−0.162016	0.486116	0.092297	0.083*	
H8B_3	−0.166159	0.368881	0.063961	0.083*	
H8C_3	−0.069602	0.433757	0.059408	0.083*	
C9_3	−0.0372 (2)	0.2519 (2)	0.11578 (17)	0.0504 (7)	
H9A_3	−0.077160	0.248981	0.071511	0.061*	
H9B_3	−0.085861	0.236230	0.142182	0.061*	
C10_3	0.0393 (3)	0.1755 (2)	0.10564 (19)	0.0597 (8)	
H10A_3	0.064973	0.164542	0.148351	0.090*	
H10B_3	0.096768	0.198221	0.089218	0.090*	
H10C_3	0.005959	0.114054	0.072723	0.090*	
N1_4	0.44069 (16)	1.08429 (16)	−0.04471 (11)	0.0384 (5)	
H1_4	0.487319	1.105894	−0.062794	0.046*	
N2_4	0.40331 (15)	0.99038 (16)	−0.06630 (11)	0.0367 (5)	
N3_4	0.44049 (15)	0.93620 (16)	−0.11519 (11)	0.0387 (5)	
N4_4	0.43051 (15)	1.23836 (16)	0.03830 (11)	0.0387 (5)	
N5_4	0.32823 (15)	1.11255 (17)	0.03422 (12)	0.0416 (5)	
H5_4	0.298817	1.052399	0.020607	0.050*	
N6_4	0.30723 (16)	1.18724 (17)	0.08510 (12)	0.0454 (5)	
N7_4	0.37655 (18)	1.35449 (18)	0.13303 (13)	0.0508 (6)	
N8_4	0.41790 (16)	0.76781 (17)	−0.18395 (11)	0.0394 (5)	
N9_4	0.33162 (15)	0.80481 (16)	−0.10139 (11)	0.0400 (5)	
H9_4	0.308221	0.840643	−0.066084	0.048*	
N10_4	0.30752 (16)	0.70805 (16)	−0.13165 (12)	0.0419 (5)	
N11_4	0.35635 (18)	0.59420 (18)	−0.22857 (13)	0.0475 (6)	
C1_4	0.40172 (16)	1.14436 (19)	0.00721 (13)	0.0347 (5)	
C2_4	0.36988 (18)	1.2590 (2)	0.08435 (14)	0.0400 (6)	
C3_4	0.39662 (17)	0.83937 (19)	−0.13265 (13)	0.0367 (5)	
C4_4	0.36125 (19)	0.6915 (2)	−0.18002 (13)	0.0391 (6)	
O1_4	0.3177 (2)	1.36992 (17)	0.17246 (14)	0.0772 (8)	
O2_4	0.44388 (17)	1.41447 (16)	0.13289 (12)	0.0633 (6)	
O3_4	0.30323 (17)	0.52957 (16)	−0.22050 (12)	0.0634 (6)	
O4_4	0.4056 (2)	0.58404 (17)	−0.27334 (11)	0.0644 (6)	
N12_4	0.20162 (18)	0.90173 (19)	0.04309 (13)	0.0487 (6)	
O5_4	0.25448 (18)	0.92206 (16)	0.00533 (12)	0.0636 (6)	
O6_4	0.1838 (2)	0.81661 (19)	0.04000 (14)	0.0829 (8)	
O7_4	0.1651 (3)	0.9697 (2)	0.08166 (17)	0.0990 (10)	
N13_4	0.0330 (9)	0.8640 (7)	0.1444 (7)	0.0446 (16)	0.374 (3)
H13_4	0.053493	0.856709	0.100267	0.054*	0.374 (3)
C5_4	−0.0339 (12)	0.9494 (10)	0.1549 (8)	0.045 (2)	0.374 (3)
H5A_4	0.011335	1.010955	0.169747	0.054*	0.374 (3)
H5B_4	−0.070496	0.950329	0.191847	0.054*	0.374 (3)
C6_4	−0.1129 (14)	0.9493 (14)	0.0945 (7)	0.048 (3)	0.374 (3)
H6A_4	−0.173305	0.905656	0.090284	0.072*	0.374 (3)
H6B_4	−0.083003	0.926173	0.053968	0.072*	0.374 (3)
H6C_4	−0.133386	1.016087	0.100294	0.072*	0.374 (3)
C7_4	−0.0250 (8)	0.7695 (7)	0.1339 (5)	0.0479 (19)	0.374 (3)
H7A_4	−0.047353	0.775265	0.176809	0.057*	0.374 (3)
H7B_4	−0.088316	0.766732	0.100946	0.057*	0.374 (3)
C8_4	0.0138 (14)	0.6741 (10)	0.1126 (12)	0.075 (4)	0.374 (3)
H8A_4	−0.043342	0.623274	0.099190	0.112*	0.374 (3)
H8B_4	0.063273	0.665539	0.149690	0.112*	0.374 (3)
H8C_4	0.047835	0.668690	0.074448	0.112*	0.374 (3)
C9_4	0.1334 (7)	0.8835 (8)	0.1935 (5)	0.067 (2)	0.374 (3)
H9A_4	0.176785	0.827936	0.180750	0.080*	0.374 (3)
H9B_4	0.171402	0.943926	0.194142	0.080*	0.374 (3)
C10_4	0.1086 (11)	0.8946 (10)	0.2576 (6)	0.082 (3)	0.374 (3)
H10A_4	0.052191	0.846223	0.252914	0.123*	0.374 (3)
H10B_4	0.087492	0.960907	0.276497	0.123*	0.374 (3)
H10C_4	0.169022	0.884375	0.287513	0.123*	0.374 (3)
N13B_4	−0.0317 (7)	0.8594 (6)	0.1597 (4)	0.0504 (15)	0.307 (3)
H13B_4	−0.100953	0.831891	0.161133	0.060*	0.307 (3)
C5B_4	0.0383 (8)	0.8723 (8)	0.2281 (5)	0.0584 (19)	0.307 (3)
H5C_4	0.016772	0.929409	0.261527	0.070*	0.307 (3)
H5D_4	0.023398	0.813792	0.241076	0.070*	0.307 (3)
C6B_4	0.1540 (10)	0.8866 (10)	0.2354 (7)	0.071 (3)	0.307 (3)
H6D_4	0.171567	0.944677	0.223461	0.106*	0.307 (3)
H6E_4	0.178670	0.828651	0.205421	0.106*	0.307 (3)
H6F_4	0.186623	0.895725	0.281850	0.106*	0.307 (3)
C7B_4	−0.0457 (18)	0.9514 (11)	0.1441 (9)	0.045 (2)	0.307 (3)
H7C_4	0.022460	0.986542	0.150993	0.054*	0.307 (3)
H7D_4	−0.085624	0.993709	0.175484	0.054*	0.307 (3)
C8B_4	−0.100 (2)	0.935 (2)	0.0739 (10)	0.060 (4)	0.307 (3)
H8D_4	−0.156413	0.883721	0.062215	0.090*	0.307 (3)
H8E_4	−0.051510	0.914107	0.043201	0.090*	0.307 (3)
H8F_4	−0.127584	0.995983	0.070224	0.090*	0.307 (3)
C9B_4	0.0106 (10)	0.7847 (8)	0.1063 (6)	0.062 (2)	0.307 (3)
H9C_4	0.070851	0.816080	0.096606	0.075*	0.307 (3)
H9D_4	−0.042155	0.763629	0.064752	0.075*	0.307 (3)
C10B_4	0.0431 (16)	0.6943 (10)	0.1248 (10)	0.060 (4)	0.307 (3)
H10D_4	0.086476	0.657919	0.094153	0.090*	0.307 (3)
H10E_4	−0.018118	0.652158	0.121356	0.090*	0.307 (3)
H10F_4	0.081899	0.714727	0.170941	0.090*	0.307 (3)
N13C_4	0.0348 (11)	0.8469 (9)	0.1473 (7)	0.0451 (17)	0.318 (3)
H13C_4	0.082297	0.858973	0.118439	0.054*	0.318 (3)
C5C_4	−0.0460 (12)	0.9206 (10)	0.1532 (8)	0.049 (2)	0.318 (3)
H5E_4	−0.096652	0.904735	0.178228	0.059*	0.318 (3)
H5F_4	−0.012831	0.987087	0.178767	0.059*	0.318 (3)
C6C_4	−0.101 (2)	0.920 (2)	0.0853 (12)	0.057 (4)	0.318 (3)
H6G_4	−0.133110	0.854343	0.059636	0.085*	0.318 (3)
H6H_4	−0.051550	0.939251	0.061287	0.085*	0.318 (3)
H6I_4	−0.154015	0.967105	0.090963	0.085*	0.318 (3)
C7C_4	−0.0267 (10)	0.7492 (9)	0.1120 (7)	0.051 (2)	0.318 (3)
H7E_4	−0.076570	0.738683	0.139350	0.061*	0.318 (3)
H7F_4	−0.065711	0.749198	0.068178	0.061*	0.318 (3)
C8C_4	0.0431 (15)	0.6688 (13)	0.1017 (11)	0.060 (3)	0.318 (3)
H8G_4	0.004252	0.607114	0.073034	0.090*	0.318 (3)
H8H_4	0.073210	0.662070	0.145146	0.090*	0.318 (3)
H8I_4	0.098126	0.683980	0.080131	0.090*	0.318 (3)
C9C_4	0.0970 (9)	0.8364 (9)	0.2104 (6)	0.067 (2)	0.318 (3)
H9E_4	0.050669	0.816501	0.236561	0.080*	0.318 (3)
H9F_4	0.145269	0.784929	0.198618	0.080*	0.318 (3)
C10C_4	0.1551 (14)	0.9309 (11)	0.2511 (9)	0.091 (4)	0.318 (3)
H10G_4	0.107815	0.983039	0.258742	0.136*	0.318 (3)
H10H_4	0.206911	0.946353	0.227276	0.136*	0.318 (3)
H10I_4	0.189265	0.926076	0.294307	0.136*	0.318 (3)

### 5,5'-(Triaz-1-ene-1,3-diyl)bis(3-nitro-1H-1,2,4-triazole)–triethylammonium nitrate (1/1) Atomic displacement parameters (Å^2^)

**Table:**

	*U*^11^	*U*^22^	*U*^33^	*U*^12^	*U*^13^	*U*^23^
N1_1	0.0346 (10)	0.0353 (12)	0.0364 (11)	0.0116 (8)	0.0099 (9)	0.0071 (9)
N2_1	0.0306 (9)	0.0331 (11)	0.0333 (11)	0.0054 (8)	0.0054 (8)	0.0041 (8)
N3_1	0.0346 (10)	0.0344 (11)	0.0365 (11)	0.0100 (8)	0.0055 (9)	0.0061 (9)
N4_1	0.0276 (9)	0.0448 (13)	0.0382 (12)	0.0103 (8)	0.0061 (8)	0.0105 (9)
N5_1	0.0287 (9)	0.0478 (13)	0.0415 (12)	0.0131 (9)	0.0092 (9)	0.0140 (10)
N6_1	0.0330 (10)	0.0575 (15)	0.0436 (13)	0.0150 (10)	0.0116 (9)	0.0182 (11)
N7_1	0.0399 (12)	0.0732 (18)	0.0526 (15)	0.0240 (12)	0.0183 (11)	0.0299 (13)
N8_1	0.0350 (10)	0.0383 (12)	0.0347 (11)	0.0099 (8)	0.0092 (9)	0.0067 (9)
N9_1	0.0374 (10)	0.0360 (12)	0.0385 (12)	0.0132 (8)	0.0140 (9)	0.0095 (9)
N10_1	0.0382 (11)	0.0401 (13)	0.0460 (13)	0.0125 (9)	0.0135 (10)	0.0117 (10)
N11_1	0.0426 (11)	0.0427 (13)	0.0426 (13)	0.0080 (10)	0.0088 (10)	0.0127 (10)
C1_1	0.0250 (10)	0.0346 (13)	0.0369 (13)	0.0050 (9)	0.0038 (9)	0.0044 (10)
C2_1	0.0267 (10)	0.0526 (16)	0.0405 (14)	0.0105 (10)	0.0070 (10)	0.0166 (12)
C3_1	0.0310 (11)	0.0325 (13)	0.0332 (13)	0.0076 (9)	0.0071 (10)	0.0026 (10)
C4_1	0.0344 (11)	0.0353 (14)	0.0369 (13)	0.0076 (10)	0.0074 (10)	0.0062 (10)
O1_1	0.0547 (12)	0.0852 (17)	0.0589 (14)	0.0395 (11)	0.0235 (10)	0.0386 (12)
O2_1	0.0782 (16)	0.126 (2)	0.0856 (18)	0.0631 (16)	0.0565 (15)	0.0700 (17)
O3_1	0.0925 (17)	0.0591 (14)	0.0520 (13)	0.0229 (12)	0.0354 (13)	0.0231 (11)
O4_1	0.0516 (11)	0.0469 (12)	0.0584 (13)	0.0162 (9)	0.0073 (10)	0.0214 (10)
N12_1	0.0522 (13)	0.0388 (14)	0.0504 (15)	0.0013 (10)	0.0241 (11)	0.0036 (11)
O5_1	0.0652 (12)	0.0572 (13)	0.0538 (12)	0.0349 (10)	0.0317 (10)	0.0213 (10)
O6_1	0.0804 (16)	0.0407 (13)	0.098 (2)	0.0141 (11)	0.0365 (15)	0.0105 (12)
O7_1	0.200 (4)	0.092 (2)	0.0688 (19)	−0.002 (2)	0.082 (2)	0.0113 (16)
N13_1	0.035 (2)	0.045 (2)	0.0338 (19)	0.0102 (17)	0.0133 (18)	0.0116 (17)
C5_1	0.049 (3)	0.062 (3)	0.043 (3)	0.003 (3)	0.005 (2)	0.005 (3)
C6_1	0.054 (4)	0.107 (6)	0.053 (5)	−0.010 (5)	0.012 (4)	0.027 (5)
C7_1	0.045 (2)	0.049 (3)	0.042 (3)	0.005 (3)	0.014 (2)	0.019 (2)
C8_1	0.056 (5)	0.040 (4)	0.067 (5)	−0.001 (3)	0.006 (4)	0.022 (4)
C9_1	0.038 (2)	0.035 (3)	0.045 (3)	0.012 (2)	0.017 (2)	0.017 (2)
C10_1	0.044 (3)	0.033 (3)	0.080 (4)	0.011 (2)	0.014 (3)	0.012 (3)
N13B_1	0.030 (2)	0.040 (2)	0.037 (2)	0.0073 (19)	0.0118 (19)	0.010 (2)
C5B_1	0.041 (3)	0.038 (3)	0.048 (3)	0.011 (2)	0.004 (2)	0.012 (2)
C6B_1	0.069 (5)	0.048 (5)	0.056 (5)	−0.006 (4)	0.004 (4)	0.024 (4)
C7B_1	0.033 (3)	0.038 (3)	0.041 (3)	0.008 (2)	0.021 (2)	0.008 (2)
C8B_1	0.043 (4)	0.036 (5)	0.037 (5)	0.007 (4)	0.010 (4)	0.003 (4)
C9B_1	0.032 (3)	0.053 (4)	0.042 (3)	0.009 (3)	0.008 (3)	0.007 (3)
C10B_1	0.040 (3)	0.050 (4)	0.036 (4)	0.007 (3)	0.015 (3)	0.010 (3)
N13C_1	0.038 (3)	0.043 (3)	0.040 (3)	0.008 (3)	0.012 (3)	0.012 (3)
C5C_1	0.040 (4)	0.046 (4)	0.044 (4)	0.008 (4)	0.005 (4)	0.013 (4)
C6C_1	0.041 (7)	0.063 (8)	0.038 (7)	0.007 (8)	0.013 (6)	0.004 (7)
C7C_1	0.037 (4)	0.040 (4)	0.041 (4)	0.005 (4)	0.021 (4)	0.012 (4)
C8C_1	0.041 (9)	0.040 (9)	0.050 (9)	0.003 (8)	0.011 (8)	0.015 (8)
C9C_1	0.040 (4)	0.043 (4)	0.050 (4)	0.012 (4)	0.012 (4)	0.015 (4)
C10C_1	0.057 (9)	0.031 (8)	0.067 (9)	0.004 (8)	0.009 (8)	0.020 (7)
N1_2	0.0332 (10)	0.0390 (12)	0.0353 (11)	0.0096 (8)	0.0081 (9)	0.0111 (9)
N2_2	0.0289 (9)	0.0376 (12)	0.0332 (11)	0.0035 (8)	0.0034 (8)	0.0084 (9)
N3_2	0.0358 (10)	0.0396 (12)	0.0359 (12)	0.0052 (8)	0.0065 (9)	0.0083 (9)
N4_2	0.0274 (9)	0.0392 (12)	0.0374 (11)	0.0083 (8)	0.0082 (8)	0.0112 (9)
N5_2	0.0343 (10)	0.0488 (13)	0.0472 (13)	0.0170 (9)	0.0163 (9)	0.0207 (10)
N6_2	0.0366 (11)	0.0517 (14)	0.0563 (14)	0.0180 (10)	0.0218 (10)	0.0256 (11)
N7_2	0.0376 (11)	0.0563 (15)	0.0576 (15)	0.0196 (10)	0.0251 (11)	0.0284 (12)
N8_2	0.0401 (11)	0.0520 (14)	0.0339 (12)	−0.0018 (10)	0.0024 (9)	0.0131 (10)
N9_2	0.0345 (10)	0.0425 (13)	0.0410 (12)	0.0047 (9)	0.0088 (9)	0.0165 (10)
N10_2	0.0356 (10)	0.0450 (13)	0.0520 (14)	0.0032 (9)	0.0045 (10)	0.0226 (11)
N11_2	0.0492 (13)	0.0650 (18)	0.0520 (16)	−0.0094 (12)	−0.0026 (12)	0.0321 (13)
C1_2	0.0254 (10)	0.0348 (13)	0.0374 (13)	0.0052 (9)	0.0055 (9)	0.0083 (10)
C2_2	0.0270 (10)	0.0453 (15)	0.0488 (16)	0.0107 (10)	0.0135 (10)	0.0191 (12)
C3_2	0.0304 (11)	0.0428 (15)	0.0333 (13)	−0.0021 (10)	0.0020 (10)	0.0077 (10)
C4_2	0.0362 (12)	0.0478 (16)	0.0407 (15)	−0.0072 (11)	−0.0039 (11)	0.0178 (12)
O1_2	0.0649 (13)	0.0601 (14)	0.0655 (14)	0.0341 (11)	0.0348 (11)	0.0367 (11)
O2_2	0.0715 (14)	0.0854 (17)	0.0681 (15)	0.0419 (13)	0.0492 (13)	0.0432 (13)
O3_2	0.119 (2)	0.0734 (17)	0.0539 (15)	−0.0083 (15)	0.0230 (15)	0.0307 (13)
O4_2	0.0556 (13)	0.101 (2)	0.099 (2)	0.0173 (13)	0.0058 (13)	0.0722 (17)
N12_2	0.0394 (11)	0.0332 (12)	0.0488 (13)	0.0107 (9)	0.0124 (10)	0.0110 (10)
O5_2	0.0536 (11)	0.0543 (13)	0.0625 (13)	0.0291 (9)	0.0325 (10)	0.0284 (10)
O6_2	0.0637 (13)	0.0515 (13)	0.0805 (16)	0.0272 (10)	0.0209 (12)	0.0310 (12)
O7_2	0.0726 (15)	0.0708 (16)	0.0896 (18)	0.0297 (12)	0.0510 (14)	0.0424 (14)
N13_2	0.0375 (11)	0.0349 (12)	0.0720 (17)	0.0096 (9)	0.0225 (11)	0.0150 (11)
C5_2	0.0410 (14)	0.0518 (19)	0.086 (2)	0.0040 (13)	0.0179 (15)	0.0283 (17)
C6_2	0.077 (2)	0.065 (2)	0.074 (2)	−0.0016 (18)	0.017 (2)	0.0193 (19)
C7_2	0.0435 (14)	0.0425 (16)	0.073 (2)	0.0004 (12)	0.0264 (14)	0.0151 (14)
C8_2	0.0475 (16)	0.0489 (19)	0.089 (3)	0.0013 (13)	0.0141 (16)	0.0173 (17)
C9_2	0.0413 (14)	0.0407 (17)	0.089 (2)	0.0137 (12)	0.0151 (15)	0.0181 (16)
C10_2	0.062 (2)	0.057 (2)	0.141 (4)	0.0161 (17)	0.021 (2)	0.056 (2)
N1_3	0.0358 (10)	0.0583 (15)	0.0476 (14)	−0.0060 (10)	0.0066 (10)	0.0298 (11)
N2_3	0.0315 (10)	0.0582 (15)	0.0451 (13)	−0.0040 (9)	0.0019 (9)	0.0290 (11)
N3_3	0.0329 (10)	0.0616 (15)	0.0487 (14)	−0.0059 (10)	0.0047 (10)	0.0320 (12)
N4_3	0.0322 (10)	0.0618 (16)	0.0499 (14)	−0.0036 (10)	0.0093 (10)	0.0246 (12)
N5_3	0.0394 (11)	0.0642 (17)	0.0557 (15)	−0.0065 (11)	0.0153 (11)	0.0257 (13)
N6_3	0.0442 (12)	0.0648 (17)	0.0604 (16)	−0.0051 (11)	0.0196 (12)	0.0247 (13)
N7_3	0.0539 (14)	0.0701 (19)	0.0599 (17)	−0.0105 (13)	0.0213 (13)	0.0196 (14)
N8_3	0.0374 (11)	0.0616 (16)	0.0484 (14)	−0.0034 (10)	0.0094 (10)	0.0270 (12)
N9_3	0.0490 (13)	0.0593 (16)	0.0471 (14)	−0.0101 (11)	0.0165 (11)	0.0227 (12)
N10_3	0.0492 (13)	0.0653 (18)	0.0518 (15)	−0.0118 (12)	0.0163 (12)	0.0211 (13)
N11_3	0.0406 (12)	0.0695 (18)	0.0495 (15)	−0.0043 (11)	0.0080 (11)	0.0228 (13)
C1_3	0.0282 (11)	0.0578 (18)	0.0554 (17)	−0.0030 (11)	0.0059 (11)	0.0326 (14)
C2_3	0.0346 (13)	0.066 (2)	0.0521 (17)	−0.0039 (12)	0.0121 (12)	0.0252 (15)
C3_3	0.0327 (12)	0.0636 (19)	0.0415 (15)	−0.0061 (11)	0.0036 (11)	0.0293 (14)
C4_3	0.0360 (13)	0.068 (2)	0.0480 (17)	−0.0045 (12)	0.0072 (12)	0.0290 (15)
O1_3	0.0911 (18)	0.096 (2)	0.0613 (15)	−0.0204 (15)	0.0379 (14)	0.0169 (14)
O2_3	0.0734 (15)	0.0684 (16)	0.0707 (16)	−0.0230 (12)	0.0284 (13)	0.0179 (12)
O3_3	0.0697 (14)	0.0760 (17)	0.0613 (15)	−0.0302 (13)	0.0108 (12)	0.0132 (12)
O4_3	0.0821 (16)	0.0715 (16)	0.0759 (17)	0.0240 (13)	0.0465 (14)	0.0333 (13)
N12_3	0.0429 (12)	0.0592 (16)	0.0599 (16)	0.0023 (11)	0.0192 (11)	0.0353 (13)
O5_3	0.0796 (16)	0.0700 (16)	0.0761 (16)	−0.0151 (12)	0.0490 (14)	0.0192 (13)
O6_3	0.0810 (16)	0.0642 (16)	0.101 (2)	−0.0041 (13)	0.0344 (15)	0.0453 (15)
O7_3	0.0737 (16)	0.0825 (19)	0.099 (2)	0.0089 (14)	0.0528 (16)	0.0206 (16)
N13_3	0.0361 (10)	0.0414 (12)	0.0379 (12)	0.0003 (9)	0.0113 (9)	0.0171 (9)
C5_3	0.067 (2)	0.105 (3)	0.047 (2)	0.003 (2)	0.0034 (17)	0.0349 (19)
C6_3	0.060 (2)	0.150 (5)	0.055 (2)	0.018 (2)	0.0049 (18)	−0.021 (3)
C7_3	0.0465 (14)	0.0437 (17)	0.0593 (19)	0.0076 (12)	0.0206 (14)	0.0099 (13)
C8_3	0.0448 (15)	0.0477 (18)	0.076 (2)	0.0093 (13)	0.0060 (15)	0.0273 (16)
C9_3	0.0439 (14)	0.0377 (15)	0.075 (2)	0.0001 (11)	0.0171 (14)	0.0231 (14)
C10_3	0.0618 (18)	0.0447 (18)	0.084 (2)	0.0073 (14)	0.0255 (17)	0.0304 (17)
N1_4	0.0356 (10)	0.0427 (13)	0.0399 (12)	−0.0029 (9)	0.0095 (9)	0.0163 (10)
N2_4	0.0300 (9)	0.0396 (12)	0.0415 (12)	−0.0013 (8)	0.0017 (9)	0.0171 (9)
N3_4	0.0339 (10)	0.0394 (12)	0.0431 (12)	−0.0016 (8)	0.0051 (9)	0.0148 (10)
N4_4	0.0327 (10)	0.0429 (13)	0.0428 (12)	−0.0006 (9)	0.0097 (9)	0.0156 (10)
N5_4	0.0307 (10)	0.0413 (13)	0.0534 (14)	−0.0013 (9)	0.0112 (9)	0.0147 (10)
N6_4	0.0350 (10)	0.0437 (13)	0.0587 (15)	−0.0006 (9)	0.0182 (10)	0.0135 (11)
N7_4	0.0441 (12)	0.0487 (15)	0.0618 (16)	−0.0005 (10)	0.0255 (12)	0.0132 (12)
N8_4	0.0376 (10)	0.0443 (13)	0.0367 (12)	0.0020 (9)	0.0077 (9)	0.0133 (10)
N9_4	0.0351 (10)	0.0414 (13)	0.0461 (13)	0.0032 (9)	0.0117 (9)	0.0156 (10)
N10_4	0.0384 (11)	0.0390 (13)	0.0483 (13)	0.0025 (9)	0.0081 (10)	0.0142 (10)
N11_4	0.0433 (12)	0.0443 (14)	0.0506 (15)	0.0023 (10)	0.0019 (11)	0.0128 (11)
C1_4	0.0246 (10)	0.0427 (14)	0.0398 (14)	−0.0010 (9)	0.0031 (9)	0.0192 (11)
C2_4	0.0304 (11)	0.0417 (15)	0.0486 (16)	0.0022 (10)	0.0117 (11)	0.0133 (12)
C3_4	0.0294 (11)	0.0452 (15)	0.0380 (14)	0.0019 (10)	0.0037 (10)	0.0184 (11)
C4_4	0.0352 (12)	0.0418 (15)	0.0390 (14)	0.0041 (10)	0.0025 (11)	0.0132 (11)
O1_4	0.0787 (16)	0.0569 (15)	0.0968 (19)	−0.0040 (12)	0.0603 (15)	0.0035 (13)
O2_4	0.0640 (13)	0.0501 (13)	0.0722 (15)	−0.0165 (10)	0.0335 (12)	0.0051 (11)
O3_4	0.0535 (12)	0.0487 (13)	0.0742 (16)	−0.0084 (10)	0.0066 (11)	0.0039 (11)
O4_4	0.0932 (17)	0.0520 (14)	0.0519 (13)	0.0139 (12)	0.0306 (13)	0.0128 (10)
N12_4	0.0477 (13)	0.0485 (15)	0.0520 (15)	−0.0070 (11)	0.0101 (11)	0.0194 (12)
O5_4	0.0754 (14)	0.0459 (13)	0.0796 (16)	−0.0005 (10)	0.0471 (13)	0.0177 (11)
O6_4	0.108 (2)	0.0593 (16)	0.0888 (19)	−0.0183 (14)	0.0105 (16)	0.0393 (14)
O7_4	0.111 (2)	0.0744 (19)	0.114 (2)	−0.0064 (16)	0.073 (2)	0.0078 (17)
N13_4	0.042 (2)	0.045 (3)	0.050 (3)	−0.004 (3)	0.004 (2)	0.023 (2)
C5_4	0.056 (4)	0.041 (4)	0.046 (4)	−0.003 (3)	0.006 (3)	0.029 (3)
C6_4	0.061 (5)	0.056 (7)	0.032 (5)	−0.015 (5)	0.004 (5)	0.025 (5)
C7_4	0.045 (3)	0.040 (4)	0.058 (4)	0.001 (3)	0.017 (3)	0.012 (3)
C8_4	0.101 (8)	0.047 (6)	0.078 (7)	−0.002 (6)	0.034 (7)	0.013 (6)
C9_4	0.072 (4)	0.057 (4)	0.064 (4)	−0.007 (3)	−0.001 (4)	0.018 (3)
C10_4	0.094 (7)	0.057 (6)	0.080 (6)	0.029 (5)	0.030 (6)	−0.008 (5)
N13B_4	0.060 (3)	0.043 (3)	0.052 (3)	−0.001 (3)	0.005 (3)	0.024 (2)
C5B_4	0.079 (4)	0.048 (4)	0.045 (4)	0.024 (3)	0.002 (3)	0.014 (3)
C6B_4	0.132 (8)	0.036 (6)	0.038 (6)	−0.030 (6)	0.009 (6)	0.010 (5)
C7B_4	0.052 (4)	0.045 (4)	0.048 (4)	0.003 (3)	0.006 (4)	0.029 (3)
C8B_4	0.071 (7)	0.068 (8)	0.044 (7)	−0.014 (7)	−0.002 (6)	0.031 (6)
C9B_4	0.069 (4)	0.056 (4)	0.063 (4)	−0.008 (4)	0.014 (4)	0.020 (3)
C10B_4	0.070 (6)	0.022 (5)	0.081 (8)	−0.014 (5)	0.037 (6)	−0.005 (5)
N13C_4	0.045 (3)	0.050 (3)	0.046 (3)	−0.011 (3)	0.004 (2)	0.027 (3)
C5C_4	0.052 (4)	0.055 (5)	0.047 (4)	−0.006 (4)	0.010 (3)	0.028 (4)
C6C_4	0.063 (6)	0.065 (8)	0.050 (8)	−0.015 (6)	0.012 (6)	0.030 (6)
C7C_4	0.048 (3)	0.046 (4)	0.060 (4)	−0.010 (3)	0.015 (4)	0.017 (4)
C8C_4	0.055 (6)	0.046 (7)	0.086 (8)	0.001 (6)	0.027 (7)	0.024 (6)
C9C_4	0.070 (4)	0.070 (4)	0.060 (4)	−0.006 (4)	0.004 (4)	0.024 (3)
C10C_4	0.095 (7)	0.073 (8)	0.071 (7)	−0.014 (7)	−0.009 (6)	−0.012 (7)

### 5,5'-(Triaz-1-ene-1,3-diyl)bis(3-nitro-1H-1,2,4-triazole)–triethylammonium nitrate (1/1) Geometric parameters (Å, º)

**Table:**

N1_1—N2_1	1.333 (3)	N1_3—N2_3	1.339 (3)
N1_1—C1_1	1.376 (3)	N1_3—C1_3	1.367 (4)
N1_1—H1_1	0.8800	N1_3—H1_3	0.8800
N2_1—N3_1	1.272 (3)	N2_3—N3_3	1.273 (3)
N3_1—C3_1	1.404 (3)	N3_3—C3_3	1.407 (4)
N4_1—C1_1	1.317 (3)	N4_3—C1_3	1.323 (4)
N4_1—C2_1	1.347 (3)	N4_3—C2_3	1.347 (3)
N5_1—C1_1	1.343 (3)	N5_3—N6_3	1.345 (4)
N5_1—N6_1	1.352 (3)	N5_3—C1_3	1.351 (3)
N5_1—H5_1	0.8800	N5_3—H5_3	0.8800
N6_1—C2_1	1.301 (3)	N6_3—C2_3	1.312 (4)
N7_1—O2_1	1.213 (3)	N7_3—O2_3	1.227 (3)
N7_1—O1_1	1.220 (3)	N7_3—O1_3	1.237 (3)
N7_1—C2_1	1.444 (3)	N7_3—C2_3	1.435 (4)
N8_1—C3_1	1.325 (3)	N8_3—C3_3	1.313 (4)
N8_1—C4_1	1.337 (3)	N8_3—C4_3	1.348 (4)
N9_1—N10_1	1.349 (3)	N9_3—N10_3	1.344 (4)
N9_1—C3_1	1.350 (3)	N9_3—C3_3	1.353 (3)
N9_1—H9_1	0.8800	N9_3—H9_3	0.8800
N10_1—C4_1	1.314 (3)	N10_3—C4_3	1.316 (4)
N11_1—O3_1	1.219 (3)	N11_3—O4_3	1.210 (3)
N11_1—O4_1	1.223 (3)	N11_3—O3_3	1.236 (3)
N11_1—C4_1	1.453 (3)	N11_3—C4_3	1.449 (4)
N12_1—O7_1	1.220 (3)	N12_3—O6_3	1.221 (3)
N12_1—O6_1	1.228 (3)	N12_3—O7_3	1.224 (4)
N12_1—O5_1	1.245 (3)	N12_3—O5_3	1.257 (3)
N13_1—C9_1	1.504 (7)	N13_3—C9_3	1.491 (3)
N13_1—C7_1	1.518 (7)	N13_3—C5_3	1.499 (4)
N13_1—C5_1	1.520 (7)	N13_3—C7_3	1.501 (3)
N13_1—H13_1	1.0000	N13_3—H13_3	1.0000
C5_1—C6_1	1.471 (9)	C5_3—C6_3	1.478 (6)
C5_1—H5A_1	0.9900	C5_3—H5A_3	0.9900
C5_1—H5B_1	0.9900	C5_3—H5B_3	0.9900
C6_1—H6A_1	0.9800	C6_3—H6A_3	0.9800
C6_1—H6B_1	0.9800	C6_3—H6B_3	0.9800
C6_1—H6C_1	0.9800	C6_3—H6C_3	0.9800
C7_1—C8_1	1.499 (10)	C7_3—C8_3	1.493 (4)
C7_1—H7A_1	0.9900	C7_3—H7A_3	0.9900
C7_1—H7B_1	0.9900	C7_3—H7B_3	0.9900
C8_1—H8A_1	0.9800	C8_3—H8A_3	0.9800
C8_1—H8B_1	0.9800	C8_3—H8B_3	0.9800
C8_1—H8C_1	0.9800	C8_3—H8C_3	0.9800
C9_1—C10_1	1.506 (7)	C9_3—C10_3	1.501 (4)
C9_1—H9A_1	0.9900	C9_3—H9A_3	0.9900
C9_1—H9B_1	0.9900	C9_3—H9B_3	0.9900
C10_1—H10A_1	0.9800	C10_3—H10A_3	0.9800
C10_1—H10B_1	0.9800	C10_3—H10B_3	0.9800
C10_1—H10C_1	0.9800	C10_3—H10C_3	0.9800
N13B_1—C9B_1	1.493 (8)	N1_4—N2_4	1.337 (3)
N13B_1—C7B_1	1.496 (8)	N1_4—C1_4	1.363 (3)
N13B_1—C5B_1	1.514 (7)	N1_4—H1_4	0.8800
N13B_1—H13B_1	1.0000	N2_4—N3_4	1.275 (3)
C5B_1—C6B_1	1.497 (10)	N3_4—C3_4	1.408 (3)
C5B_1—H5C_1	0.9900	N4_4—C1_4	1.321 (3)
C5B_1—H5D_1	0.9900	N4_4—C2_4	1.341 (3)
C6B_1—H6D_1	0.9800	N5_4—N6_4	1.342 (3)
C6B_1—H6E_1	0.9800	N5_4—C1_4	1.351 (3)
C6B_1—H6F_1	0.9800	N5_4—H5_4	0.8800
C7B_1—C8B_1	1.488 (11)	N6_4—C2_4	1.316 (3)
C7B_1—H7C_1	0.9900	N7_4—O1_4	1.216 (3)
C7B_1—H7D_1	0.9900	N7_4—O2_4	1.228 (3)
C8B_1—H8D_1	0.9800	N7_4—C2_4	1.431 (4)
C8B_1—H8E_1	0.9800	N8_4—C3_4	1.324 (3)
C8B_1—H8F_1	0.9800	N8_4—C4_4	1.349 (3)
C9B_1—C10B_1	1.512 (9)	N9_4—N10_4	1.344 (3)
C9B_1—H9C_1	0.9900	N9_4—C3_4	1.348 (3)
C9B_1—H9D_1	0.9900	N9_4—H9_4	0.8800
C10B_1—H10D_1	0.9800	N10_4—C4_4	1.317 (3)
C10B_1—H10E_1	0.9800	N11_4—O4_4	1.221 (3)
C10B_1—H10F_1	0.9800	N11_4—O3_4	1.225 (3)
N13C_1—C7C_1	1.503 (14)	N11_4—C4_4	1.451 (4)
N13C_1—C5C_1	1.510 (14)	N12_4—O6_4	1.221 (3)
N13C_1—C9C_1	1.511 (14)	N12_4—O7_4	1.234 (4)
N13C_1—H13C_1	1.0000	N12_4—O5_4	1.248 (3)
C5C_1—C6C_1	1.504 (15)	N13_4—C7_4	1.482 (10)
C5C_1—H5E_1	0.9900	N13_4—C9_4	1.507 (10)
C5C_1—H5F_1	0.9900	N13_4—C5_4	1.512 (12)
C6C_1—H6G_1	0.9800	N13_4—H13_4	1.0000
C6C_1—H6H_1	0.9800	C5_4—C6_4	1.516 (12)
C6C_1—H6I_1	0.9800	C5_4—H5A_4	0.9900
C7C_1—C8C_1	1.481 (15)	C5_4—H5B_4	0.9900
C7C_1—H7E_1	0.9900	C6_4—H6A_4	0.9800
C7C_1—H7F_1	0.9900	C6_4—H6B_4	0.9800
C8C_1—H8G_1	0.9800	C6_4—H6C_4	0.9800
C8C_1—H8H_1	0.9800	C7_4—C8_4	1.440 (12)
C8C_1—H8I_1	0.9800	C7_4—H7A_4	0.9900
C9C_1—C10C_1	1.494 (14)	C7_4—H7B_4	0.9900
C9C_1—H9E_1	0.9900	C8_4—H8A_4	0.9800
C9C_1—H9F_1	0.9900	C8_4—H8B_4	0.9800
C10C_1—H10G_1	0.9800	C8_4—H8C_4	0.9800
C10C_1—H10H_1	0.9800	C9_4—C10_4	1.424 (11)
C10C_1—H10I_1	0.9800	C9_4—H9A_4	0.9900
N1_2—N2_2	1.334 (3)	C9_4—H9B_4	0.9900
N1_2—C1_2	1.381 (3)	C10_4—H10A_4	0.9800
N1_2—H1_2	0.8800	C10_4—H10B_4	0.9800
N2_2—N3_2	1.269 (3)	C10_4—H10C_4	0.9800
N3_2—C3_2	1.401 (3)	N13B_4—C7B_4	1.471 (12)
N4_2—C1_2	1.324 (3)	N13B_4—C9B_4	1.500 (11)
N4_2—C2_2	1.350 (3)	N13B_4—C5B_4	1.546 (10)
N5_2—C1_2	1.340 (3)	N13B_4—H13B_4	1.0000
N5_2—N6_2	1.360 (3)	C5B_4—C6B_4	1.516 (13)
N5_2—H5_2	0.8800	C5B_4—H5C_4	0.9900
N6_2—C2_2	1.305 (3)	C5B_4—H5D_4	0.9900
N7_2—O1_2	1.220 (3)	C6B_4—H6D_4	0.9800
N7_2—O2_2	1.226 (3)	C6B_4—H6E_4	0.9800
N7_2—C2_2	1.446 (3)	C6B_4—H6F_4	0.9800
N8_2—C3_2	1.332 (3)	C7B_4—C8B_4	1.491 (13)
N8_2—C4_2	1.343 (4)	C7B_4—H7C_4	0.9900
N9_2—C3_2	1.342 (3)	C7B_4—H7D_4	0.9900
N9_2—N10_2	1.344 (3)	C8B_4—H8D_4	0.9800
N9_2—H9_2	0.8800	C8B_4—H8E_4	0.9800
N10_2—C4_2	1.311 (4)	C8B_4—H8F_4	0.9800
N11_2—O3_2	1.214 (4)	C9B_4—C10B_4	1.524 (14)
N11_2—O4_2	1.227 (4)	C9B_4—H9C_4	0.9900
N11_2—C4_2	1.456 (3)	C9B_4—H9D_4	0.9900
N12_2—O7_2	1.238 (3)	C10B_4—H10D_4	0.9800
N12_2—O6_2	1.241 (3)	C10B_4—H10E_4	0.9800
N12_2—O5_2	1.256 (3)	C10B_4—H10F_4	0.9800
N13_2—C5_2	1.496 (4)	N13C_4—C9C_4	1.506 (12)
N13_2—C9_2	1.502 (4)	N13C_4—C7C_4	1.513 (11)
N13_2—C7_2	1.502 (3)	N13C_4—C5C_4	1.516 (12)
N13_2—H13_2	1.0000	N13C_4—H13C_4	1.0000
C5_2—C6_2	1.480 (5)	C5C_4—C6C_4	1.505 (13)
C5_2—H5A_2	0.9900	C5C_4—H5E_4	0.9900
C5_2—H5B_2	0.9900	C5C_4—H5F_4	0.9900
C6_2—H6A_2	0.9800	C6C_4—H6G_4	0.9800
C6_2—H6B_2	0.9800	C6C_4—H6H_4	0.9800
C6_2—H6C_2	0.9800	C6C_4—H6I_4	0.9800
C7_2—C8_2	1.497 (5)	C7C_4—C8C_4	1.484 (12)
C7_2—H7A_2	0.9900	C7C_4—H7E_4	0.9900
C7_2—H7B_2	0.9900	C7C_4—H7F_4	0.9900
C8_2—H8A_2	0.9800	C8C_4—H8G_4	0.9800
C8_2—H8B_2	0.9800	C8C_4—H8H_4	0.9800
C8_2—H8C_2	0.9800	C8C_4—H8I_4	0.9800
C9_2—C10_2	1.512 (4)	C9C_4—C10C_4	1.478 (12)
C9_2—H9A_2	0.9900	C9C_4—H9E_4	0.9900
C9_2—H9B_2	0.9900	C9C_4—H9F_4	0.9900
C10_2—H10A_2	0.9800	C10C_4—H10G_4	0.9800
C10_2—H10B_2	0.9800	C10C_4—H10H_4	0.9800
C10_2—H10C_2	0.9800	C10C_4—H10I_4	0.9800
			
N2_1—N1_1—C1_1	115.87 (19)	N2_3—N1_3—C1_3	116.1 (2)
N2_1—N1_1—H1_1	122.1	N2_3—N1_3—H1_3	122.0
C1_1—N1_1—H1_1	122.1	C1_3—N1_3—H1_3	122.0
N3_1—N2_1—N1_1	114.45 (19)	N3_3—N2_3—N1_3	114.3 (2)
N2_1—N3_1—C3_1	109.35 (19)	N2_3—N3_3—C3_3	109.4 (2)
C1_1—N4_1—C2_1	100.57 (19)	C1_3—N4_3—C2_3	100.4 (2)
C1_1—N5_1—N6_1	109.5 (2)	N6_3—N5_3—C1_3	110.0 (2)
C1_1—N5_1—H5_1	125.3	N6_3—N5_3—H5_3	125.0
N6_1—N5_1—H5_1	125.3	C1_3—N5_3—H5_3	125.0
C2_1—N6_1—N5_1	101.09 (19)	C2_3—N6_3—N5_3	100.7 (2)
O2_1—N7_1—O1_1	124.5 (2)	O2_3—N7_3—O1_3	124.1 (3)
O2_1—N7_1—C2_1	118.6 (2)	O2_3—N7_3—C2_3	116.5 (2)
O1_1—N7_1—C2_1	117.0 (2)	O1_3—N7_3—C2_3	119.3 (3)
C3_1—N8_1—C4_1	101.08 (19)	C3_3—N8_3—C4_3	101.3 (2)
N10_1—N9_1—C3_1	109.8 (2)	N10_3—N9_3—C3_3	110.5 (2)
N10_1—N9_1—H9_1	125.1	N10_3—N9_3—H9_3	124.8
C3_1—N9_1—H9_1	125.1	C3_3—N9_3—H9_3	124.8
C4_1—N10_1—N9_1	100.98 (19)	C4_3—N10_3—N9_3	100.5 (2)
O3_1—N11_1—O4_1	124.9 (2)	O4_3—N11_3—O3_3	124.2 (3)
O3_1—N11_1—C4_1	117.4 (2)	O4_3—N11_3—C4_3	118.1 (3)
O4_1—N11_1—C4_1	117.7 (2)	O3_3—N11_3—C4_3	117.7 (2)
N4_1—C1_1—N5_1	111.1 (2)	N4_3—C1_3—N5_3	110.8 (3)
N4_1—C1_1—N1_1	125.8 (2)	N4_3—C1_3—N1_3	125.6 (2)
N5_1—C1_1—N1_1	123.0 (2)	N5_3—C1_3—N1_3	123.5 (3)
N6_1—C2_1—N4_1	117.8 (2)	N6_3—C2_3—N4_3	118.1 (3)
N6_1—C2_1—N7_1	121.2 (2)	N6_3—C2_3—N7_3	121.2 (3)
N4_1—C2_1—N7_1	120.9 (2)	N4_3—C2_3—N7_3	120.7 (2)
N8_1—C3_1—N9_1	110.5 (2)	N8_3—C3_3—N9_3	110.2 (3)
N8_1—C3_1—N3_1	122.9 (2)	N8_3—C3_3—N3_3	123.3 (2)
N9_1—C3_1—N3_1	126.6 (2)	N9_3—C3_3—N3_3	126.5 (3)
N10_1—C4_1—N8_1	117.7 (2)	N10_3—C4_3—N8_3	117.5 (3)
N10_1—C4_1—N11_1	119.6 (2)	N10_3—C4_3—N11_3	119.2 (2)
N8_1—C4_1—N11_1	122.7 (2)	N8_3—C4_3—N11_3	123.3 (3)
O7_1—N12_1—O6_1	121.3 (3)	O6_3—N12_3—O7_3	120.4 (2)
O7_1—N12_1—O5_1	118.6 (3)	O6_3—N12_3—O5_3	119.8 (3)
O6_1—N12_1—O5_1	120.1 (3)	O7_3—N12_3—O5_3	119.8 (3)
C9_1—N13_1—C7_1	112.9 (4)	C9_3—N13_3—C5_3	111.1 (2)
C9_1—N13_1—C5_1	109.9 (5)	C9_3—N13_3—C7_3	110.9 (2)
C7_1—N13_1—C5_1	113.1 (5)	C5_3—N13_3—C7_3	112.4 (2)
C9_1—N13_1—H13_1	106.8	C9_3—N13_3—H13_3	107.4
C7_1—N13_1—H13_1	106.8	C5_3—N13_3—H13_3	107.4
C5_1—N13_1—H13_1	106.8	C7_3—N13_3—H13_3	107.4
C6_1—C5_1—N13_1	112.2 (6)	C6_3—C5_3—N13_3	114.2 (3)
C6_1—C5_1—H5A_1	109.2	C6_3—C5_3—H5A_3	108.7
N13_1—C5_1—H5A_1	109.2	N13_3—C5_3—H5A_3	108.7
C6_1—C5_1—H5B_1	109.2	C6_3—C5_3—H5B_3	108.7
N13_1—C5_1—H5B_1	109.2	N13_3—C5_3—H5B_3	108.7
H5A_1—C5_1—H5B_1	107.9	H5A_3—C5_3—H5B_3	107.6
C5_1—C6_1—H6A_1	109.5	C5_3—C6_3—H6A_3	109.5
C5_1—C6_1—H6B_1	109.5	C5_3—C6_3—H6B_3	109.5
H6A_1—C6_1—H6B_1	109.5	H6A_3—C6_3—H6B_3	109.5
C5_1—C6_1—H6C_1	109.5	C5_3—C6_3—H6C_3	109.5
H6A_1—C6_1—H6C_1	109.5	H6A_3—C6_3—H6C_3	109.5
H6B_1—C6_1—H6C_1	109.5	H6B_3—C6_3—H6C_3	109.5
C8_1—C7_1—N13_1	111.9 (6)	C8_3—C7_3—N13_3	114.2 (2)
C8_1—C7_1—H7A_1	109.2	C8_3—C7_3—H7A_3	108.7
N13_1—C7_1—H7A_1	109.2	N13_3—C7_3—H7A_3	108.7
C8_1—C7_1—H7B_1	109.2	C8_3—C7_3—H7B_3	108.7
N13_1—C7_1—H7B_1	109.2	N13_3—C7_3—H7B_3	108.7
H7A_1—C7_1—H7B_1	107.9	H7A_3—C7_3—H7B_3	107.6
C7_1—C8_1—H8A_1	109.5	C7_3—C8_3—H8A_3	109.5
C7_1—C8_1—H8B_1	109.5	C7_3—C8_3—H8B_3	109.5
H8A_1—C8_1—H8B_1	109.5	H8A_3—C8_3—H8B_3	109.5
C7_1—C8_1—H8C_1	109.5	C7_3—C8_3—H8C_3	109.5
H8A_1—C8_1—H8C_1	109.5	H8A_3—C8_3—H8C_3	109.5
H8B_1—C8_1—H8C_1	109.5	H8B_3—C8_3—H8C_3	109.5
N13_1—C9_1—C10_1	112.4 (5)	N13_3—C9_3—C10_3	113.5 (2)
N13_1—C9_1—H9A_1	109.1	N13_3—C9_3—H9A_3	108.9
C10_1—C9_1—H9A_1	109.1	C10_3—C9_3—H9A_3	108.9
N13_1—C9_1—H9B_1	109.1	N13_3—C9_3—H9B_3	108.9
C10_1—C9_1—H9B_1	109.1	C10_3—C9_3—H9B_3	108.9
H9A_1—C9_1—H9B_1	107.8	H9A_3—C9_3—H9B_3	107.7
C9_1—C10_1—H10A_1	109.5	C9_3—C10_3—H10A_3	109.5
C9_1—C10_1—H10B_1	109.5	C9_3—C10_3—H10B_3	109.5
H10A_1—C10_1—H10B_1	109.5	H10A_3—C10_3—H10B_3	109.5
C9_1—C10_1—H10C_1	109.5	C9_3—C10_3—H10C_3	109.5
H10A_1—C10_1—H10C_1	109.5	H10A_3—C10_3—H10C_3	109.5
H10B_1—C10_1—H10C_1	109.5	H10B_3—C10_3—H10C_3	109.5
C9B_1—N13B_1—C7B_1	109.4 (5)	N2_4—N1_4—C1_4	115.50 (19)
C9B_1—N13B_1—C5B_1	111.2 (6)	N2_4—N1_4—H1_4	122.2
C7B_1—N13B_1—C5B_1	112.7 (5)	C1_4—N1_4—H1_4	122.2
C9B_1—N13B_1—H13B_1	107.7	N3_4—N2_4—N1_4	114.09 (19)
C7B_1—N13B_1—H13B_1	107.7	N2_4—N3_4—C3_4	108.78 (19)
C5B_1—N13B_1—H13B_1	107.7	C1_4—N4_4—C2_4	100.4 (2)
C6B_1—C5B_1—N13B_1	113.9 (6)	N6_4—N5_4—C1_4	109.2 (2)
C6B_1—C5B_1—H5C_1	108.8	N6_4—N5_4—H5_4	125.4
N13B_1—C5B_1—H5C_1	108.8	C1_4—N5_4—H5_4	125.4
C6B_1—C5B_1—H5D_1	108.8	C2_4—N6_4—N5_4	101.4 (2)
N13B_1—C5B_1—H5D_1	108.8	O1_4—N7_4—O2_4	124.7 (3)
H5C_1—C5B_1—H5D_1	107.7	O1_4—N7_4—C2_4	118.4 (2)
C5B_1—C6B_1—H6D_1	109.5	O2_4—N7_4—C2_4	116.9 (2)
C5B_1—C6B_1—H6E_1	109.5	C3_4—N8_4—C4_4	100.5 (2)
H6D_1—C6B_1—H6E_1	109.5	N10_4—N9_4—C3_4	110.6 (2)
C5B_1—C6B_1—H6F_1	109.5	N10_4—N9_4—H9_4	124.7
H6D_1—C6B_1—H6F_1	109.5	C3_4—N9_4—H9_4	124.7
H6E_1—C6B_1—H6F_1	109.5	C4_4—N10_4—N9_4	100.4 (2)
C8B_1—C7B_1—N13B_1	114.3 (7)	O4_4—N11_4—O3_4	126.0 (3)
C8B_1—C7B_1—H7C_1	108.7	O4_4—N11_4—C4_4	117.3 (2)
N13B_1—C7B_1—H7C_1	108.7	O3_4—N11_4—C4_4	116.6 (2)
C8B_1—C7B_1—H7D_1	108.7	N4_4—C1_4—N5_4	111.3 (2)
N13B_1—C7B_1—H7D_1	108.7	N4_4—C1_4—N1_4	125.9 (2)
H7C_1—C7B_1—H7D_1	107.6	N5_4—C1_4—N1_4	122.8 (2)
C7B_1—C8B_1—H8D_1	109.5	N6_4—C2_4—N4_4	117.6 (2)
C7B_1—C8B_1—H8E_1	109.5	N6_4—C2_4—N7_4	121.1 (2)
H8D_1—C8B_1—H8E_1	109.5	N4_4—C2_4—N7_4	121.2 (2)
C7B_1—C8B_1—H8F_1	109.5	N8_4—C3_4—N9_4	110.6 (2)
H8D_1—C8B_1—H8F_1	109.5	N8_4—C3_4—N3_4	122.0 (2)
H8E_1—C8B_1—H8F_1	109.5	N9_4—C3_4—N3_4	127.5 (2)
N13B_1—C9B_1—C10B_1	113.8 (6)	N10_4—C4_4—N8_4	118.0 (2)
N13B_1—C9B_1—H9C_1	108.8	N10_4—C4_4—N11_4	120.4 (2)
C10B_1—C9B_1—H9C_1	108.8	N8_4—C4_4—N11_4	121.5 (2)
N13B_1—C9B_1—H9D_1	108.8	O6_4—N12_4—O7_4	121.3 (3)
C10B_1—C9B_1—H9D_1	108.8	O6_4—N12_4—O5_4	120.6 (3)
H9C_1—C9B_1—H9D_1	107.7	O7_4—N12_4—O5_4	118.0 (3)
C9B_1—C10B_1—H10D_1	109.5	C7_4—N13_4—C9_4	114.9 (8)
C9B_1—C10B_1—H10E_1	109.5	C7_4—N13_4—C5_4	112.8 (10)
H10D_1—C10B_1—H10E_1	109.5	C9_4—N13_4—C5_4	114.5 (9)
C9B_1—C10B_1—H10F_1	109.5	C7_4—N13_4—H13_4	104.4
H10D_1—C10B_1—H10F_1	109.5	C9_4—N13_4—H13_4	104.4
H10E_1—C10B_1—H10F_1	109.5	C5_4—N13_4—H13_4	104.4
C7C_1—N13C_1—C5C_1	112.8 (16)	N13_4—C5_4—C6_4	116.0 (11)
C7C_1—N13C_1—C9C_1	108.5 (15)	N13_4—C5_4—H5A_4	108.3
C5C_1—N13C_1—C9C_1	112.7 (16)	C6_4—C5_4—H5A_4	108.3
C7C_1—N13C_1—H13C_1	107.5	N13_4—C5_4—H5B_4	108.3
C5C_1—N13C_1—H13C_1	107.5	C6_4—C5_4—H5B_4	108.3
C9C_1—N13C_1—H13C_1	107.5	H5A_4—C5_4—H5B_4	107.4
C6C_1—C5C_1—N13C_1	114.8 (17)	C5_4—C6_4—H6A_4	109.5
C6C_1—C5C_1—H5E_1	108.6	C5_4—C6_4—H6B_4	109.5
N13C_1—C5C_1—H5E_1	108.6	H6A_4—C6_4—H6B_4	109.5
C6C_1—C5C_1—H5F_1	108.6	C5_4—C6_4—H6C_4	109.5
N13C_1—C5C_1—H5F_1	108.6	H6A_4—C6_4—H6C_4	109.5
H5E_1—C5C_1—H5F_1	107.5	H6B_4—C6_4—H6C_4	109.5
C5C_1—C6C_1—H6G_1	109.5	C8_4—C7_4—N13_4	125.4 (11)
C5C_1—C6C_1—H6H_1	109.5	C8_4—C7_4—H7A_4	106.0
H6G_1—C6C_1—H6H_1	109.5	N13_4—C7_4—H7A_4	106.0
C5C_1—C6C_1—H6I_1	109.5	C8_4—C7_4—H7B_4	106.0
H6G_1—C6C_1—H6I_1	109.5	N13_4—C7_4—H7B_4	106.0
H6H_1—C6C_1—H6I_1	109.5	H7A_4—C7_4—H7B_4	106.3
C8C_1—C7C_1—N13C_1	115.4 (19)	C7_4—C8_4—H8A_4	109.5
C8C_1—C7C_1—H7E_1	108.4	C7_4—C8_4—H8B_4	109.5
N13C_1—C7C_1—H7E_1	108.4	H8A_4—C8_4—H8B_4	109.5
C8C_1—C7C_1—H7F_1	108.4	C7_4—C8_4—H8C_4	109.5
N13C_1—C7C_1—H7F_1	108.4	H8A_4—C8_4—H8C_4	109.5
H7E_1—C7C_1—H7F_1	107.5	H8B_4—C8_4—H8C_4	109.5
C7C_1—C8C_1—H8G_1	109.5	C10_4—C9_4—N13_4	106.7 (10)
C7C_1—C8C_1—H8H_1	109.5	C10_4—C9_4—H9A_4	110.4
H8G_1—C8C_1—H8H_1	109.5	N13_4—C9_4—H9A_4	110.4
C7C_1—C8C_1—H8I_1	109.5	C10_4—C9_4—H9B_4	110.4
H8G_1—C8C_1—H8I_1	109.5	N13_4—C9_4—H9B_4	110.4
H8H_1—C8C_1—H8I_1	109.5	H9A_4—C9_4—H9B_4	108.6
C10C_1—C9C_1—N13C_1	112.1 (15)	C9_4—C10_4—H10A_4	109.5
C10C_1—C9C_1—H9E_1	109.2	C9_4—C10_4—H10B_4	109.5
N13C_1—C9C_1—H9E_1	109.2	H10A_4—C10_4—H10B_4	109.5
C10C_1—C9C_1—H9F_1	109.2	C9_4—C10_4—H10C_4	109.5
N13C_1—C9C_1—H9F_1	109.2	H10A_4—C10_4—H10C_4	109.5
H9E_1—C9C_1—H9F_1	107.9	H10B_4—C10_4—H10C_4	109.5
C9C_1—C10C_1—H10G_1	109.5	C7B_4—N13B_4—C9B_4	111.5 (10)
C9C_1—C10C_1—H10H_1	109.5	C7B_4—N13B_4—C5B_4	114.1 (10)
H10G_1—C10C_1—H10H_1	109.5	C9B_4—N13B_4—C5B_4	108.4 (8)
C9C_1—C10C_1—H10I_1	109.5	C7B_4—N13B_4—H13B_4	107.5
H10G_1—C10C_1—H10I_1	109.5	C9B_4—N13B_4—H13B_4	107.5
H10H_1—C10C_1—H10I_1	109.5	C5B_4—N13B_4—H13B_4	107.5
N2_2—N1_2—C1_2	115.66 (19)	C6B_4—C5B_4—N13B_4	119.8 (9)
N2_2—N1_2—H1_2	122.2	C6B_4—C5B_4—H5C_4	107.4
C1_2—N1_2—H1_2	122.2	N13B_4—C5B_4—H5C_4	107.4
N3_2—N2_2—N1_2	113.78 (19)	C6B_4—C5B_4—H5D_4	107.4
N2_2—N3_2—C3_2	109.4 (2)	N13B_4—C5B_4—H5D_4	107.4
C1_2—N4_2—C2_2	100.00 (19)	H5C_4—C5B_4—H5D_4	106.9
C1_2—N5_2—N6_2	109.5 (2)	C5B_4—C6B_4—H6D_4	109.5
C1_2—N5_2—H5_2	125.3	C5B_4—C6B_4—H6E_4	109.5
N6_2—N5_2—H5_2	125.3	H6D_4—C6B_4—H6E_4	109.5
C2_2—N6_2—N5_2	100.7 (2)	C5B_4—C6B_4—H6F_4	109.5
O1_2—N7_2—O2_2	124.3 (2)	H6D_4—C6B_4—H6F_4	109.5
O1_2—N7_2—C2_2	116.8 (2)	H6E_4—C6B_4—H6F_4	109.5
O2_2—N7_2—C2_2	118.8 (2)	N13B_4—C7B_4—C8B_4	112.7 (15)
C3_2—N8_2—C4_2	100.6 (2)	N13B_4—C7B_4—H7C_4	109.0
C3_2—N9_2—N10_2	110.0 (2)	C8B_4—C7B_4—H7C_4	109.0
C3_2—N9_2—H9_2	125.0	N13B_4—C7B_4—H7D_4	109.0
N10_2—N9_2—H9_2	125.0	C8B_4—C7B_4—H7D_4	109.0
C4_2—N10_2—N9_2	101.3 (2)	H7C_4—C7B_4—H7D_4	107.8
O3_2—N11_2—O4_2	124.7 (3)	C7B_4—C8B_4—H8D_4	109.5
O3_2—N11_2—C4_2	118.0 (3)	C7B_4—C8B_4—H8E_4	109.5
O4_2—N11_2—C4_2	117.3 (3)	H8D_4—C8B_4—H8E_4	109.5
N4_2—C1_2—N5_2	111.5 (2)	C7B_4—C8B_4—H8F_4	109.5
N4_2—C1_2—N1_2	125.2 (2)	H8D_4—C8B_4—H8F_4	109.5
N5_2—C1_2—N1_2	123.3 (2)	H8E_4—C8B_4—H8F_4	109.5
N6_2—C2_2—N4_2	118.3 (2)	N13B_4—C9B_4—C10B_4	114.0 (11)
N6_2—C2_2—N7_2	120.9 (2)	N13B_4—C9B_4—H9C_4	108.8
N4_2—C2_2—N7_2	120.7 (2)	C10B_4—C9B_4—H9C_4	108.8
N8_2—C3_2—N9_2	110.6 (2)	N13B_4—C9B_4—H9D_4	108.8
N8_2—C3_2—N3_2	122.4 (2)	C10B_4—C9B_4—H9D_4	108.8
N9_2—C3_2—N3_2	127.0 (2)	H9C_4—C9B_4—H9D_4	107.7
N10_2—C4_2—N8_2	117.5 (2)	C9B_4—C10B_4—H10D_4	109.5
N10_2—C4_2—N11_2	120.3 (3)	C9B_4—C10B_4—H10E_4	109.5
N8_2—C4_2—N11_2	122.2 (3)	H10D_4—C10B_4—H10E_4	109.5
O7_2—N12_2—O6_2	121.2 (2)	C9B_4—C10B_4—H10F_4	109.5
O7_2—N12_2—O5_2	119.6 (2)	H10D_4—C10B_4—H10F_4	109.5
O6_2—N12_2—O5_2	119.2 (2)	H10E_4—C10B_4—H10F_4	109.5
C5_2—N13_2—C9_2	114.0 (2)	C9C_4—N13C_4—C7C_4	105.4 (9)
C5_2—N13_2—C7_2	112.2 (2)	C9C_4—N13C_4—C5C_4	119.0 (12)
C9_2—N13_2—C7_2	111.9 (2)	C7C_4—N13C_4—C5C_4	103.6 (10)
C5_2—N13_2—H13_2	106.0	C9C_4—N13C_4—H13C_4	109.4
C9_2—N13_2—H13_2	106.0	C7C_4—N13C_4—H13C_4	109.4
C7_2—N13_2—H13_2	106.0	C5C_4—N13C_4—H13C_4	109.4
C6_2—C5_2—N13_2	113.7 (3)	C6C_4—C5C_4—N13C_4	111.7 (14)
C6_2—C5_2—H5A_2	108.8	C6C_4—C5C_4—H5E_4	109.3
N13_2—C5_2—H5A_2	108.8	N13C_4—C5C_4—H5E_4	109.3
C6_2—C5_2—H5B_2	108.8	C6C_4—C5C_4—H5F_4	109.3
N13_2—C5_2—H5B_2	108.8	N13C_4—C5C_4—H5F_4	109.3
H5A_2—C5_2—H5B_2	107.7	H5E_4—C5C_4—H5F_4	107.9
C5_2—C6_2—H6A_2	109.5	C5C_4—C6C_4—H6G_4	109.5
C5_2—C6_2—H6B_2	109.5	C5C_4—C6C_4—H6H_4	109.5
H6A_2—C6_2—H6B_2	109.5	H6G_4—C6C_4—H6H_4	109.5
C5_2—C6_2—H6C_2	109.5	C5C_4—C6C_4—H6I_4	109.5
H6A_2—C6_2—H6C_2	109.5	H6G_4—C6C_4—H6I_4	109.5
H6B_2—C6_2—H6C_2	109.5	H6H_4—C6C_4—H6I_4	109.5
C8_2—C7_2—N13_2	112.1 (3)	C8C_4—C7C_4—N13C_4	109.9 (12)
C8_2—C7_2—H7A_2	109.2	C8C_4—C7C_4—H7E_4	109.7
N13_2—C7_2—H7A_2	109.2	N13C_4—C7C_4—H7E_4	109.7
C8_2—C7_2—H7B_2	109.2	C8C_4—C7C_4—H7F_4	109.7
N13_2—C7_2—H7B_2	109.2	N13C_4—C7C_4—H7F_4	109.7
H7A_2—C7_2—H7B_2	107.9	H7E_4—C7C_4—H7F_4	108.2
C7_2—C8_2—H8A_2	109.5	C7C_4—C8C_4—H8G_4	109.5
C7_2—C8_2—H8B_2	109.5	C7C_4—C8C_4—H8H_4	109.5
H8A_2—C8_2—H8B_2	109.5	H8G_4—C8C_4—H8H_4	109.5
C7_2—C8_2—H8C_2	109.5	C7C_4—C8C_4—H8I_4	109.5
H8A_2—C8_2—H8C_2	109.5	H8G_4—C8C_4—H8I_4	109.5
H8B_2—C8_2—H8C_2	109.5	H8H_4—C8C_4—H8I_4	109.5
N13_2—C9_2—C10_2	112.9 (2)	C10C_4—C9C_4—N13C_4	109.7 (11)
N13_2—C9_2—H9A_2	109.0	C10C_4—C9C_4—H9E_4	109.7
C10_2—C9_2—H9A_2	109.0	N13C_4—C9C_4—H9E_4	109.7
N13_2—C9_2—H9B_2	109.0	C10C_4—C9C_4—H9F_4	109.7
C10_2—C9_2—H9B_2	109.0	N13C_4—C9C_4—H9F_4	109.7
H9A_2—C9_2—H9B_2	107.8	H9E_4—C9C_4—H9F_4	108.2
C9_2—C10_2—H10A_2	109.5	C9C_4—C10C_4—H10G_4	109.5
C9_2—C10_2—H10B_2	109.5	C9C_4—C10C_4—H10H_4	109.5
H10A_2—C10_2—H10B_2	109.5	H10G_4—C10C_4—H10H_4	109.5
C9_2—C10_2—H10C_2	109.5	C9C_4—C10C_4—H10I_4	109.5
H10A_2—C10_2—H10C_2	109.5	H10G_4—C10C_4—H10I_4	109.5
H10B_2—C10_2—H10C_2	109.5	H10H_4—C10C_4—H10I_4	109.5
			
C1_1—N1_1—N2_1—N3_1	−179.4 (2)	C1_3—N1_3—N2_3—N3_3	−178.7 (2)
N1_1—N2_1—N3_1—C3_1	179.36 (19)	N1_3—N2_3—N3_3—C3_3	178.7 (2)
C1_1—N5_1—N6_1—C2_1	0.3 (3)	C1_3—N5_3—N6_3—C2_3	0.8 (3)
C3_1—N9_1—N10_1—C4_1	−0.5 (3)	C3_3—N9_3—N10_3—C4_3	−1.0 (3)
C2_1—N4_1—C1_1—N5_1	0.2 (3)	C2_3—N4_3—C1_3—N5_3	1.3 (3)
C2_1—N4_1—C1_1—N1_1	−178.3 (2)	C2_3—N4_3—C1_3—N1_3	−176.5 (3)
N6_1—N5_1—C1_1—N4_1	−0.3 (3)	N6_3—N5_3—C1_3—N4_3	−1.4 (3)
N6_1—N5_1—C1_1—N1_1	178.2 (2)	N6_3—N5_3—C1_3—N1_3	176.4 (2)
N2_1—N1_1—C1_1—N4_1	174.8 (2)	N2_3—N1_3—C1_3—N4_3	175.0 (2)
N2_1—N1_1—C1_1—N5_1	−3.5 (3)	N2_3—N1_3—C1_3—N5_3	−2.5 (4)
N5_1—N6_1—C2_1—N4_1	−0.2 (3)	N5_3—N6_3—C2_3—N4_3	0.1 (3)
N5_1—N6_1—C2_1—N7_1	−176.8 (2)	N5_3—N6_3—C2_3—N7_3	−178.0 (3)
C1_1—N4_1—C2_1—N6_1	0.0 (3)	C1_3—N4_3—C2_3—N6_3	−0.8 (3)
C1_1—N4_1—C2_1—N7_1	176.6 (2)	C1_3—N4_3—C2_3—N7_3	177.2 (3)
O2_1—N7_1—C2_1—N6_1	−4.3 (4)	O2_3—N7_3—C2_3—N6_3	−174.3 (3)
O1_1—N7_1—C2_1—N6_1	175.1 (3)	O1_3—N7_3—C2_3—N6_3	7.7 (5)
O2_1—N7_1—C2_1—N4_1	179.2 (3)	O2_3—N7_3—C2_3—N4_3	7.7 (4)
O1_1—N7_1—C2_1—N4_1	−1.4 (4)	O1_3—N7_3—C2_3—N4_3	−170.3 (3)
C4_1—N8_1—C3_1—N9_1	0.5 (3)	C4_3—N8_3—C3_3—N9_3	−0.4 (3)
C4_1—N8_1—C3_1—N3_1	178.0 (2)	C4_3—N8_3—C3_3—N3_3	178.1 (2)
N10_1—N9_1—C3_1—N8_1	−0.1 (3)	N10_3—N9_3—C3_3—N8_3	0.9 (3)
N10_1—N9_1—C3_1—N3_1	−177.4 (2)	N10_3—N9_3—C3_3—N3_3	−177.6 (2)
N2_1—N3_1—C3_1—N8_1	179.2 (2)	N2_3—N3_3—C3_3—N8_3	−178.3 (2)
N2_1—N3_1—C3_1—N9_1	−3.7 (3)	N2_3—N3_3—C3_3—N9_3	0.0 (4)
N9_1—N10_1—C4_1—N8_1	0.9 (3)	N9_3—N10_3—C4_3—N8_3	0.8 (3)
N9_1—N10_1—C4_1—N11_1	−178.4 (2)	N9_3—N10_3—C4_3—N11_3	−179.4 (2)
C3_1—N8_1—C4_1—N10_1	−0.9 (3)	C3_3—N8_3—C4_3—N10_3	−0.2 (3)
C3_1—N8_1—C4_1—N11_1	178.4 (2)	C3_3—N8_3—C4_3—N11_3	179.9 (3)
O3_1—N11_1—C4_1—N10_1	167.5 (3)	O4_3—N11_3—C4_3—N10_3	172.4 (3)
O4_1—N11_1—C4_1—N10_1	−12.4 (4)	O3_3—N11_3—C4_3—N10_3	−8.9 (4)
O3_1—N11_1—C4_1—N8_1	−11.7 (4)	O4_3—N11_3—C4_3—N8_3	−7.7 (4)
O4_1—N11_1—C4_1—N8_1	168.4 (2)	O3_3—N11_3—C4_3—N8_3	171.0 (3)
C9_1—N13_1—C5_1—C6_1	169.7 (6)	C9_3—N13_3—C5_3—C6_3	172.5 (3)
C7_1—N13_1—C5_1—C6_1	−63.0 (7)	C7_3—N13_3—C5_3—C6_3	−62.5 (4)
C9_1—N13_1—C7_1—C8_1	−58.1 (8)	C9_3—N13_3—C7_3—C8_3	−61.9 (3)
C5_1—N13_1—C7_1—C8_1	176.2 (7)	C5_3—N13_3—C7_3—C8_3	173.0 (3)
C7_1—N13_1—C9_1—C10_1	−62.6 (7)	C5_3—N13_3—C9_3—C10_3	−59.6 (3)
C5_1—N13_1—C9_1—C10_1	64.8 (6)	C7_3—N13_3—C9_3—C10_3	174.6 (3)
C9B_1—N13B_1—C5B_1—C6B_1	−59.1 (8)	C1_4—N1_4—N2_4—N3_4	179.4 (2)
C7B_1—N13B_1—C5B_1—C6B_1	64.3 (8)	N1_4—N2_4—N3_4—C3_4	179.13 (19)
C9B_1—N13B_1—C7B_1—C8B_1	−176.7 (9)	C1_4—N5_4—N6_4—C2_4	0.4 (3)
C5B_1—N13B_1—C7B_1—C8B_1	58.9 (10)	C3_4—N9_4—N10_4—C4_4	0.0 (3)
C7B_1—N13B_1—C9B_1—C10B_1	−177.7 (7)	C2_4—N4_4—C1_4—N5_4	−0.1 (3)
C5B_1—N13B_1—C9B_1—C10B_1	−52.5 (9)	C2_4—N4_4—C1_4—N1_4	−178.9 (2)
C7C_1—N13C_1—C5C_1—C6C_1	−37 (3)	N6_4—N5_4—C1_4—N4_4	−0.2 (3)
C9C_1—N13C_1—C5C_1—C6C_1	−161 (2)	N6_4—N5_4—C1_4—N1_4	178.6 (2)
C5C_1—N13C_1—C7C_1—C8C_1	172 (3)	N2_4—N1_4—C1_4—N4_4	176.9 (2)
C9C_1—N13C_1—C7C_1—C8C_1	−62 (4)	N2_4—N1_4—C1_4—N5_4	−1.7 (3)
C7C_1—N13C_1—C9C_1—C10C_1	171 (2)	N5_4—N6_4—C2_4—N4_4	−0.6 (3)
C5C_1—N13C_1—C9C_1—C10C_1	−64 (3)	N5_4—N6_4—C2_4—N7_4	−177.6 (2)
C1_2—N1_2—N2_2—N3_2	−178.0 (2)	C1_4—N4_4—C2_4—N6_4	0.5 (3)
N1_2—N2_2—N3_2—C3_2	179.67 (19)	C1_4—N4_4—C2_4—N7_4	177.5 (2)
C1_2—N5_2—N6_2—C2_2	0.5 (3)	O1_4—N7_4—C2_4—N6_4	−5.0 (4)
C3_2—N9_2—N10_2—C4_2	−0.7 (3)	O2_4—N7_4—C2_4—N6_4	173.1 (3)
C2_2—N4_2—C1_2—N5_2	1.0 (3)	O1_4—N7_4—C2_4—N4_4	178.0 (3)
C2_2—N4_2—C1_2—N1_2	−177.3 (2)	O2_4—N7_4—C2_4—N4_4	−3.9 (4)
N6_2—N5_2—C1_2—N4_2	−1.0 (3)	C4_4—N8_4—C3_4—N9_4	0.2 (3)
N6_2—N5_2—C1_2—N1_2	177.3 (2)	C4_4—N8_4—C3_4—N3_4	179.0 (2)
N2_2—N1_2—C1_2—N4_2	176.0 (2)	N10_4—N9_4—C3_4—N8_4	−0.1 (3)
N2_2—N1_2—C1_2—N5_2	−2.0 (3)	N10_4—N9_4—C3_4—N3_4	−178.8 (2)
N5_2—N6_2—C2_2—N4_2	0.2 (3)	N2_4—N3_4—C3_4—N8_4	177.0 (2)
N5_2—N6_2—C2_2—N7_2	−177.7 (2)	N2_4—N3_4—C3_4—N9_4	−4.4 (3)
C1_2—N4_2—C2_2—N6_2	−0.7 (3)	N9_4—N10_4—C4_4—N8_4	0.2 (3)
C1_2—N4_2—C2_2—N7_2	177.2 (2)	N9_4—N10_4—C4_4—N11_4	−177.9 (2)
O1_2—N7_2—C2_2—N6_2	−177.7 (3)	C3_4—N8_4—C4_4—N10_4	−0.2 (3)
O2_2—N7_2—C2_2—N6_2	4.5 (4)	C3_4—N8_4—C4_4—N11_4	177.8 (2)
O1_2—N7_2—C2_2—N4_2	4.5 (4)	O4_4—N11_4—C4_4—N10_4	178.0 (2)
O2_2—N7_2—C2_2—N4_2	−173.4 (3)	O3_4—N11_4—C4_4—N10_4	−2.4 (4)
C4_2—N8_2—C3_2—N9_2	−0.3 (3)	O4_4—N11_4—C4_4—N8_4	0.0 (4)
C4_2—N8_2—C3_2—N3_2	179.5 (2)	O3_4—N11_4—C4_4—N8_4	179.6 (2)
N10_2—N9_2—C3_2—N8_2	0.7 (3)	C7_4—N13_4—C5_4—C6_4	−68.3 (19)
N10_2—N9_2—C3_2—N3_2	−179.1 (2)	C9_4—N13_4—C5_4—C6_4	157.8 (14)
N2_2—N3_2—C3_2—N8_2	178.7 (2)	C9_4—N13_4—C7_4—C8_4	−56 (2)
N2_2—N3_2—C3_2—N9_2	−1.6 (3)	C5_4—N13_4—C7_4—C8_4	170.0 (16)
N9_2—N10_2—C4_2—N8_2	0.5 (3)	C7_4—N13_4—C9_4—C10_4	−66.4 (13)
N9_2—N10_2—C4_2—N11_2	−178.8 (2)	C5_4—N13_4—C9_4—C10_4	66.5 (14)
C3_2—N8_2—C4_2—N10_2	−0.2 (3)	C7B_4—N13B_4—C5B_4—C6B_4	−77.1 (15)
C3_2—N8_2—C4_2—N11_2	179.2 (2)	C9B_4—N13B_4—C5B_4—C6B_4	47.8 (13)
O3_2—N11_2—C4_2—N10_2	175.8 (3)	C9B_4—N13B_4—C7B_4—C8B_4	48 (2)
O4_2—N11_2—C4_2—N10_2	−5.5 (4)	C5B_4—N13B_4—C7B_4—C8B_4	171.2 (17)
O3_2—N11_2—C4_2—N8_2	−3.5 (4)	C7B_4—N13B_4—C9B_4—C10B_4	170.6 (14)
O4_2—N11_2—C4_2—N8_2	175.2 (3)	C5B_4—N13B_4—C9B_4—C10B_4	44.2 (15)
C9_2—N13_2—C5_2—C6_2	−58.5 (3)	C9C_4—N13C_4—C5C_4—C6C_4	−179.4 (16)
C7_2—N13_2—C5_2—C6_2	70.1 (3)	C7C_4—N13C_4—C5C_4—C6C_4	−62.9 (17)
C5_2—N13_2—C7_2—C8_2	162.2 (2)	C9C_4—N13C_4—C7C_4—C8C_4	−55.1 (17)
C9_2—N13_2—C7_2—C8_2	−68.1 (3)	C5C_4—N13C_4—C7C_4—C8C_4	179.1 (13)
C5_2—N13_2—C9_2—C10_2	−56.0 (4)	C7C_4—N13C_4—C9C_4—C10C_4	−176.2 (14)
C7_2—N13_2—C9_2—C10_2	175.3 (3)	C5C_4—N13C_4—C9C_4—C10C_4	−60.6 (18)

### 5,5'-(Triaz-1-ene-1,3-diyl)bis(3-nitro-1H-1,2,4-triazole)–triethylammonium nitrate (1/1) Hydrogen-bond geometry (Å, º)

**Table:**

*D*—H···*A*	*D*—H	H···*A*	*D*···*A*	*D*—H···*A*
N1_1—H1_1···N4_2^i^	0.88	2.37	3.117 (3)	142
N1_1—H1_1···O1_2^i^	0.88	2.30	3.066 (3)	145
N5_1—H5_1···O5_1	0.88	1.87	2.745 (3)	173
N9_1—H9_1···O5_1	0.88	1.88	2.761 (3)	174
N13_1—H13_1···N12_1	1.00	2.60	3.565 (6)	162
N13_1—H13_1···O6_1	1.00	2.31	3.096 (6)	135
N13_1—H13_1···O7_1	1.00	2.26	3.249 (6)	169
C6_1—H6*A*_1···N8_4^ii^	0.98	2.69	3.650 (8)	168
C7_1—H7*B*_1···O4_4^ii^	0.99	2.55	3.387 (7)	142
C8_1—H8*B*_1···O6_2^iii^	0.98	2.58	3.518 (11)	161
C9_1—H9*B*_1···O6_2^iii^	0.99	2.44	3.222 (7)	136
N13*B*_1—H13*B*_1···N8_4^ii^	1.00	2.17	3.165 (6)	172
C5*B*_1—H5*C*_1···O2_3^iv^	0.99	2.64	3.629 (8)	173
C5*B*_1—H5*D*_1···N10_2^iii^	0.99	2.58	3.567 (7)	177
C7*B*_1—H7*D*_1···O6_1	0.99	2.62	3.547 (7)	156
C8*B*_1—H8*D*_1···O4_2^iii^	0.98	2.52	3.452 (11)	160
C9*B*_1—H9*D*_1···O2_2^iv^	0.99	2.53	3.146 (7)	120
C10*B*_1—H10*D*_1···O2_3^iv^	0.98	2.61	3.150 (8)	115
N13*C*_1—H13*C*_1···O6_1	1.00	2.29	3.27 (3)	167
N13*C*_1—H13*C*_1···O7_1	1.00	2.56	3.31 (2)	132
C5*C*_1—H5*F*_1···O2_2^iv^	0.99	2.55	3.32 (3)	135
C6*C*_1—H6*H*_1···N8_4^ii^	0.98	2.61	3.53 (3)	157
C6*C*_1—H6*I*_1···O7_1	0.98	2.52	3.34 (3)	141
C7*C*_1—H7*F*_1···O4_4^ii^	0.99	2.36	2.93 (2)	116
C9*C*_1—H9*F*_1···O6_2^iii^	0.99	2.33	3.20 (2)	147
N1_2—H1_2···N4_1^i^	0.88	2.35	3.096 (3)	143
N1_2—H1_2···O1_1^i^	0.88	2.27	3.035 (3)	145
N5_2—H5_2···O5_2	0.88	1.88	2.751 (3)	172
N9_2—H9_2···O5_2	0.88	1.89	2.770 (3)	177
N13_2—H13_2···N12_2	1.00	2.53	3.444 (3)	151
N13_2—H13_2···O6_2	1.00	1.96	2.933 (3)	164
N13_2—H13_2···O7_2	1.00	2.48	3.188 (3)	128
C5_2—H5*A*_2···O2_1	0.99	2.57	3.097 (4)	113
C9_2—H9*A*_2···O6_1^iii^	0.99	2.41	3.340 (5)	157
N1_3—H1_3···N4_4^v^	0.88	2.39	3.136 (3)	143
N1_3—H1_3···O2_4^v^	0.88	2.30	3.075 (3)	147
N5_3—H5_3···N12_3	0.88	2.70	3.523 (3)	157
N5_3—H5_3···O5_3	0.88	1.87	2.748 (4)	175
N9_3—H9_3···O5_3	0.88	1.86	2.743 (3)	178
N13_3—H13_3···N12_3	1.00	2.51	3.503 (3)	174
N13_3—H13_3···O6_3	1.00	2.14	3.068 (3)	153
N13_3—H13_3···O7_3	1.00	2.23	3.116 (3)	146
C5_3—H5*A*_3···O1_2^iv^	0.99	2.64	3.367 (4)	131
C7_3—H7*B*_3···O3_1^vi^	0.99	2.63	3.317 (4)	127
C9_3—H9*A*_3···O6_4^vii^	0.99	2.44	3.389 (4)	160
C9_3—H9*B*_3···O3_2^vi^	0.99	2.65	3.538 (4)	149
C10_3—H10*B*_3···O6_3	0.98	2.56	3.342 (4)	137
N1_4—H1_4···N4_3^v^	0.88	2.39	3.126 (3)	142
N1_4—H1_4···O2_3^v^	0.88	2.29	3.035 (3)	143
N5_4—H5_4···O5_4	0.88	1.86	2.732 (3)	169
N9_4—H9_4···O5_4	0.88	1.87	2.748 (3)	176
N13_4—H13_4···N12_4	1.00	2.65	3.505 (15)	144
N13_4—H13_4···O6_4	1.00	2.30	3.171 (16)	145
N13_4—H13_4···O7_4	1.00	2.34	3.019 (14)	124
C6_4—H6*A*_4···N10_3^vii^	0.98	2.59	3.52 (2)	157
C7_4—H7*A*_4···O3_1^vi^	0.99	2.63	3.410 (12)	136
C7_4—H7*B*_4···N10_3^vii^	0.99	2.68	3.394 (11)	129
C9_4—H9*A*_4···O1_3	0.99	2.62	3.203 (10)	118
C9_4—H9*B*_4···O7_4	0.99	2.53	3.096 (11)	116
C10_4—H10*A*_4···O1_1	0.98	2.59	3.158 (13)	117
C10_4—H10*B*_4···N6_2	0.98	2.54	3.219 (13)	126
N13*B*_4—H13*B*_4···N10_3^vii^	1.00	2.45	3.385 (9)	155
C5*B*_4—H5*C*_4···N8_2^i^	0.99	2.62	3.558 (11)	159
C5*B*_4—H5*D*_4···O1_1	0.99	2.54	3.286 (10)	132
C7*B*_4—H7*C*_4···O7_4	0.99	2.56	3.33 (2)	134
C7*B*_4—H7*D*_4···O3_2^i^	0.99	2.27	3.155 (17)	148
C9*B*_4—H9*C*_4···N12_4	0.99	2.70	3.666 (13)	166
C9*B*_4—H9*C*_4···O6_4	0.99	2.08	2.989 (12)	152
C9*B*_4—H9*C*_4···O7_4	0.99	2.64	3.540 (13)	151
C9*B*_4—H9*D*_4···O6_3^vii^	0.99	2.29	3.240 (13)	160
N13*C*_4—H13*C*_4···N12_4	1.00	2.63	3.626 (18)	176
N13*C*_4—H13*C*_4···O6_4	1.00	2.27	3.208 (18)	156
N13*C*_4—H13*C*_4···O7_4	1.00	2.31	3.208 (16)	149
C7*C*_4—H7*F*_4···O6_3^vii^	0.99	2.21	3.100 (13)	150
C8*C*_4—H8*I*_4···O6_4	0.98	2.63	3.48 (2)	146
C10*C*_4—H10*G*_4···O2_2	0.98	2.61	3.346 (17)	132
C10*C*_4—H10*H*_4···O1_3	0.98	2.56	3.18 (2)	122

Symmetry codes: (i) −*x*, −*y*+2, −*z*+1; (ii) −*x*+1, −*y*+1, −*z*; (iii) −*x*+1, −*y*+1, −*z*+1; (iv) *x*, *y*−1, *z*; (v) −*x*+1, −*y*+2, −*z*; (vi) −*x*, −*y*+1, −*z*+1; (vii) −*x*, −*y*+1, −*z*.
